# The etiology of Down syndrome: Maternal MCM9 polymorphisms increase risk of reduced recombination and nondisjunction of chromosome 21 during meiosis I within oocyte

**DOI:** 10.1371/journal.pgen.1009462

**Published:** 2021-03-22

**Authors:** Upamanyu Pal, Pinku Halder, Anirban Ray, Sumantra Sarkar, Supratim Datta, Papiya Ghosh, Sujay Ghosh

**Affiliations:** 1 Cytogenetics and Genomics Research Unit, Department of Zoology, University of Calcutta, Taraknath Palit Siksha Prangan (Ballygunge Science College Campus), Kolkata, West Bengal, India; 2 Department of Zoology, Bangabasi Morning College (affiliated to University of Calcutta), Kolkata, West Bengal, India; 3 Department of Paediatric Medicine, Institute of Post Graduate Medical Education and Research (IPGMER), Bhowanipore, Kolkata, West Bengal, India; 4 Department of Paediatric Medicine, Diamond Harbour Government Medical College & Hospital, Diamond Harbour, West Bengal, India; 5 Department of Zoology, Bijoykrishna Girls’ College (Affiliated to University of Calcutta), Howrah, West Bengal, India; Spelman College, UNITED STATES

## Abstract

Altered patterns of recombination on 21q have long been associated with the nondisjunction chromosome 21 within oocytes and the increased risk of having a child with Down syndrome. Unfortunately the genetic etiology of these altered patterns of recombination have yet to be elucidated. We for the first time genotyped the gene MCM9, a candidate gene for recombination regulation and DNA repair in mothers with or without children with Down syndrome. In our approach, we identified the location of recombination on the maternal chromosome 21 using short tandem repeat markers, then stratified our population by the origin of meiotic error and age at conception. We observed that twenty-five out of forty-one single nucleotide polymorphic sites within MCM9 exhibited an association with meiosis I error (N = 700), but not with meiosis II error (N = 125). This association was maternal age-independent. Several variants exhibited aprotective association with MI error, some were neutral. Maternal age stratified characterization of cases revealed that MCM9 risk variants were associated with an increased chance of reduced recombination on 21q within oocytes. The spatial distribution of single observed recombination events revealed no significant change in the location of recombination among women harbouring MCM9 risk, protective, or neutral variant. Additionally, we identified a total of six novel polymorphic variants and two novel alleles that were either risk imparting or protective against meiosis I nondisjunction. *In silico* analyses using five different programs suggest the risk variants either cause a change in protein function or may alter the splicing pattern of transcripts and disrupt the proportion of different isoforms of MCM9 products within oocytes. These observations bring us a significant step closer to understanding the molecular basis of recombination errors in chromosome 21 nondisjunction within oocytes that leads to birth of child with Down syndrome.

## Author summary

We studied MCM9 variations in the genome of women with a Down syndrome child by stratifying the women based on MCM9 genotypes, meiotic error group, and their age of conception. We identified polymorphisms are associated with reduced recombination and nondisjunction of chromosome 21 at the meiosis I stage of oogenesis in a maternal age-independent manner. But these variants do not affect the position of chiasma formation. *In Silico* analyses revealed the presence of MCM9 variants that may cause alteration in protein function due to amino acid substitution. We also identified splice variants in MCM9. We hypothesize that the polymorphisms in MCM9 predispose women to experience reduced recombination on chromosome 21 in oocytes at meiosis I, which ultimately leads to the birth of a child with Down syndrome.

## Introduction

Down syndrome [DS], the most frequent genetic form of live-born intellectual disability is caused by the presence of supernumerary chromosome 21 (Ch21). This extra copy of Ch21 originates from errors in the chromosome segregation process, known as nondisjunction (NDJ), that occurs in meiosis during parental gametogenesis. The error is far more frequent in oogenesis (~90% of all observed incidence) than spermatogenesis. The protracted period of maturation may provide an opportunity for genetic and environmental insults to accumulate within oocytes, which increases the risk of NDJ by several fold [1,2]. The incidence of maternal NDJ is more frequent during meiosis I (MI errors accounting for nearly 70% of all maternal cases) than meiosis II (MII errors) [3].

In search of risk factors associated with DS birth, researchers initially identified advanced maternal age at conception as a major challenge to healthy oocyte maturation and correct chromosome segregation [4]. Later, the recombination anomaly on Ch21q was characterized as the first molecular correlate that predisposes homologue pairs to segregate erroneously during meiosis [5]. In characterizing the interactions between maternal age and anomalous recombination patterns on Ch21q in NDJ, two independent studies on US [2], and, Indian [6] DS samples revealed that the absence of recombination as well as a single exchange within the telomeric region of 21q are maternal age-independent risk factors for Ch21 MI NDJ. In contrast, single recombination events near the centromere increases the chance of MII errors and this is maternal age-dependent. The presence and ideal location of recombination are prerequisites for proper chromosome separation. The chiasma placed at the middle of chromosome arms hold the chromatids tightly, balance the pull from the opposite poles, and ensure the correct movement of chromosomes towards opposite poles. Therefore, non-exchange chromosomes suffer from the risk of missegregation, and the cell may require surveillance and special separators to ensure correct segregation of non-exchange chromosomes, as evident in yeast [7]. The risk of NDJ increases gradually with age due to the rapid degradation of protein machinery that includes sister chromatid cohesins [8] and the surveillance system [7,9]. A chiasma located at the telomeric end of the chromosome probably links the homologues less efficiently to the spindle due to loss of cohesion and imprecisely orients the kinetochore towards the opposite pole [8,10]. In contrast, chiasma at proximity to the centromere, may cause chromosome entanglement which is somehow tolerated through MI, but ultimately causes separation of the sister chromatids to fail at MII [9,10]. All these observations intuitively suggest the presence of some maternal genetic risk factors that predispose the oocyte to experience recombination anomalies on Ch21, and subsequent separation errors, all in addition to advanced maternal age.

Very limited attempts have been taken to characterize the variations and/or mutations in the maternal genome that may increase recombination anomalies on Ch21q. The first known ‘candidate gene’ analysis was conducted on the gene PRDM9 (Chromosome 5; HGNC 13994) [11,12]. In this study, the maternal PRDM9 zinc finger binding domain was genotyped. The result revealed a higher frequency of the minor allele of the PRDM9 gene among the mothers of DS births. Even when the authors compared with the PRDM9 major A-allele, this minor allele variant displayed fewer predicted binding sites on 21q [13].

In search of new candidate genes whose polymorphisms/mutations are associated with altered patterns of recombination on Ch21in oocytes, we have now analysed the gene ‘Mini-chromosome maintenance 9’ [MCM9; Chromosome 6; HGNC: 21484; OMIM 610098; cytogenetic location 6q22.31; genomic coordinates (GRCh38): 6:118,813,454–118,935,158]. The gene encodes a member of the minichromosome maintenance (MCM) family proteins that play a pivotal role in replication and homologous recombination in humans [14,15]. Biochemically, MCM9 belongs to the class of AAA helicases (ATPases Associated with diverse cellular Activities) and carries a conserved helicase domain (the MCM family domain) of around 300 amino acids, a Zn-finger motif, and Walker A and B ATP hydrolysis motifs [16–18]. This protein forms a complex with MCM8 that is needed for homologous recombination [HR] repair induced by DNA interstrand crosslinks [ICLs]. Binding of this protein to chromatin is a prerequisite for recruiting the MCM2-7 complex at the replication origin [17,19]. It also acts as a positive regulator of chromatin licensing and DNA replication factor 1. It makes complexes with MCM8 and works downstream of Fanconi anemia proteins BCRA2/RAD51, which processess aberrant forks and advances the sister chromatid exchange [15,18,20,21]. This complex promotes DNA synthesis during homologous recombination, perhaps to promote the repair of broken replication fork [19,22].

We have selected MCM9 to characterize its polymorphisms as the underlying risk factors of recombination errors on Ch21, considering previous reports on its association with ovarian insufficiency, compromised fertility, and genomic instability in humans and model organisms. A mouse model [22] homozygous for MCM9^-^/MCM9^-^ revealed complete sterility in females with ovaries devoid of ovum and males with testes having limited sperm count. Moreover, homozygous MCM9^-^/MCM9^-^ embryonic fibroblasts exhibit growth defects and chromosomal damage. A study in human females revealed that the MCM9 c.1732+2 T>C variant alters a splice donor site and MCM9 c.394C>T p.Arg132(*) results in a predicted loss of functional MCM9. The repair of chromosome breaks was also impaired in lymphocytes from affected females. Both of these mutations led to defective DNA repair and chromosomal instability, and, exhibited an association with primary ovarian failure (POF) [20] and ovarian insufficiency [21]. A recent study [23] on a Chinese population provides evidence in favor of an association with premature ovarian insufficiency with three novel mutations in MCM9, namely, c.C1423T (p.L475F), c.T2921C (p.L974S), c.G3388A (p.A1130T). They also report their implication in inefficient DNA repair capacity in cultured cells. These findings strongly suggest the prospective implication of MCM9 mutants/polymorphic variantsin meiotic recombination anomalies that predispose to Ch21 NDJ.

We analyzed the MCM9 genotypes among the women having DS and euploid children to ask four basic questions 1) Are MCM9 variants associated with Ch21 NDJ in Indian Bengali women? 2) How do these MCM9 mutants/variants interact with maternal age at conception? 3) Do MCM9 mutations/polymorphisms associate the amount of observed recombination on Ch21q, stratified by maternal age and stage of origin of meiotic errors i.e., MI and MII? and 4) Do these mutations or variants influence the position of single observed recombinant events on Ch21q in interaction with maternal age and meiotic error types? We for the first time have scrutinized the complicated relationship among MCM9 variants, maternal age at conception, and recombination pattern and provide more insight into the genetic etiology of Ch21 NDJ in oocytes and subsequent DS birth.

## Results

### Metadata of sample populations

Table 1 shows the demographic and epidemiological details of the participating cases and control subjects. Case families were recruited randomly into the study following verification of DS among the new born child upon morphological examination by physician collaborators, and subsequent confirmation of Trisomy 21 status by classical karyotyping at the laboratory of the University of Calcutta. As most of the cases of DS birth were the first issue for the women, we do not have data on the subsequent children born to these mothers as they did not return to the hospital and were not followed up further. All of the epidemiological details, family history, and lifestyle data of the participating families were recorded into a pre-printed form after taking their written consent. Controls were also recruited randomly from the same hospitals which provided the DS samples, to maintain the maximum demographic and epidemiological similarities. All the control women had a healthy euploid baby at the time of reporting to our laboratory. Out of 1007 families whose parental and meiotic origin of NDJ error were determined unambiguously, 825 exhibited clear maternal origin of supernumerary Ch21. Among them, 700 and 125 families exhibited MI and MII origin of the extra Ch21, respectively. Following genotyping of the MCM9 reading frame, we characterized each variant from these two groups as ‘risk genotype’, ‘protective genotype’, or ‘neutral genotype’ based on their relationship with the NDJ error (definitions are given in the section ‘Polymorphism analyses’). The characterization of MCM9 polymorphisms was done blinded without knowing the meiotic outcome status of the error.

**Table 1:** Participant demographic and epidemiological details.

Criteria		DS bearing women		Control women
		MI	MII	
**Families referred initially**		**825**	**182**	**855**
**Samples available for genotyping**		**700**	**125**	**730**
**Mean maternal age at conception (all referred cases) [Year±SD]**		**30.5±4.01**	**33.01±3.6**	**28.8±4.6**
**Mean maternal age of women with MCM9 protective genotypes [Year±SD]**		**31.7±2.07**	**33.08±2.5**	**29.02±3.1**
**Mean maternal age of women with MCM9 risk genotypes [Year±SD]**		**31.9±3.4**	**34.01±4.1**	**30.2±5.2**
**Mean maternal age of women with MCM9 neutral genotypes [Year±SD]**		**30.8±2.7**	**32.06±3.2**	**28±3.4**
**Mean paternal age at conception. (all referred cases) [Year±SD]**		**31.4±2.01**	**31.9±6.2**	**33.41±3.5**
**Preconception maternal folic acid intake amount (mean±SDμm/day)**		**431.5±4.1**	**351.7±2.2**	**532.5±1.5**
**Socio-economic condition of families**	**Low(<INR30,000/Month) [Frequency]**	**227[0.32]**	**68[0.54]**	**268[0.36]**
	**Middle (INR 30,000–50,000/month) [Frequency]**	**385[0.55]**	**45[0.36]**	**357[0.49]**
	**High (INR >50,000/month) [Frequency]**	**88[0.13]**	**12[0.1]**	**105[0.15]**
**Locality**	**Kolkata metropolitan [Frequency]**	**515[0.74]**	**77[0.61]**	**522[0.71]**
	**Suburbs [Frequency]**	**131[0.19]**	**28[0.22]**	**149[0.2]**
	**Rural [Frequency]**	**54[0.07]**	**20[0.16]**	**59[0.09]**
**Religion**	**Hindu [Frequency]**	**631[0.9]**	**88[0.7]**	**663[0.9]**
	**Islam [Frequency]**	**59[0.08]**	**29[0.23]**	**49[0.06]**
	**Others [Frequency]**	**20[0.02]**	**8[0.07]**	**24[0.04]**
**Genotype status**	**MCM9-neutral type genotype [Frequency]**	**236[33.7]**	**58[46.4]**	**321[43.96]**
	**MCM9 protective genotype [Frequency]**	**166[23.7]**	**29[23.2]**	**197[27.0]**
	**MCM9 risk genotypes [Frequency]**	**298[42.6]**	**38[30.4]**	**212[29.04]**

### MCM9 variants and maternal age

The estimated mean age of women in the MI error group having MCM9 protective variants was 31.7 ± 2.07 (mean±SD) years, which is similar to the estimate of 31.9±3.4 years for the MI women with MCM9 risk variants (P = 0.6) and 30.8±2.7 years for the MI women with neutral variants (P = 0.2). The mean age of mothers in the MII error MCM9 risk variant category was 34.01±4.1 years, which is again concordant with the estimated mean age of 33.08±2.5 years for MII mothers having protective MCM9 variants (P = 0.71) and 32.06±3.2 years for mothers having neutral variants (P = 0.2). The mean age for MCM9 risk variant mothers having a euploid child was 30.2±5.2 years, which is again not statistically different from the estimates for control women carrying protective variants (29.02±3.1 years, P = 0.64) and neutral variants (28±3.4 years, P = 0.08). When we compared the mean age of the women in the MI risk variant group with the MII risk variant group, and the difference remained insignificant (P = 0.06). Moreover, the meiotic outcome groups with protective variants did not exhibit a difference in mean maternal age of conception when compared to control women having protective variants (MI protective vs. euploid protective P = 0.1; MII protective vs. euploid protective P = 0.08). As we dealt with maternal errors and maternal genotypes only, we did not analyze the paternal age and paternal genotypes owing to the lack of paternal tissue samples.

### MCM9 variants and their association with maternal MI and MII errors

The paternal and maternal origin of the supernumerary Ch21 was determined by genotyping a set of STR makers spanning from the centromere to the telomere of Ch21q. A subset of pericentromeric STR makers was used to interpret the meiotic stage of origin of errors. Out of 1007 families whose parental and meiotic origin of NDJ error was determined unambiguously, 825 exhibited clear maternal origin of the supernumerary Ch21. We found 700 and 125 families where the error had occurred at MI and MII, respectively. Following genotyping for the MCM9 reading frame by dideoxy sequencing, we stratified these groups again into three genotypic classes; MCM9 risk variants, MCM9 protective variants, and MCM9 neutral variants. We found an excessive number of women from the MI error group having MCM9 risk genotypes when all minor alleles for all the polymorphic sites were considered together. Women who carried one or more MCM9 risk variants represented~42.6% of the total MI error cases, in contrast to ~30.4% from the MII group and ~29% from the controls. This distribution of risk genotypes between MI and the control group was significantly different (OR = 1.84, 95% CI = 1.028–3.318, P = 0.04) but not between MII and controls (OR = 1.04, 95% CI = 0.571–1.927, P = 0.8). Though the frequency of risk variants was much higher in the MI group than in the MII group, we identified no significant difference between them (OR = 1.67, 95% CI = 0.982–3.152, P = 0.057). The statistical outcome was calculated by Fisher’s exact test.

### Polymorphism analysis

We identified 41 single nucleotide variants (SNPs) through sequencing the entire reading frame of the maternal MCM9 gene. The data on maternal genotypes and their risk association for MI and MII group are represented in Tables 2 and 3. All the polymorphic variants are present as three different genotypes in the population, namely homozygous for the major allele, homozygous for minor allele, and heterozygous. The major allele was more frequent in the population in contrast to the minor allele which probably originated from the former one by random mutation. Depending on the association of detected variants in the MI, MII, and control groups, we classified them as ‘neutral variants’, ‘risk variants’, and ‘protective variants’. Polymorphisms which showed no significant association either with cases or with controls were termed as ‘neutral variants’; the variants (minor alleles) that exhibited a significant association with NDJ were termed as ‘risk variants’, and variants that exhibited an association with controls were considered as ‘protective variants’. We calculated the odds ratio for each of the maternal genotypes for all of the detected variants, treating the homozygous major allele genotype as the reference. Of the forty-one polymorphic sites that were detected, twenty-five variants (thirty-eight genotypes) revealed risk association with MI NDJ errors. On the contrary, eight variants (thirteen genotypes) were revealed as ‘protective’ (i.e., significantly more frequent among the control women). The remainder of the eight variants did not reveal any significant association either with MI or control women, hence, ‘neutral’.The case-control analyses involving MII error women revealed a significant risk association for only one polymorphic site.

**Table 2:** Distribution of MCM9 genotypes among the control mothers (N = 730) and mothers ofchildren with Down syndrome (N = 700) who experienced meiosis I errors. The risk variants exhibited a strong association with MI errors among DS mothers and protective variants exhibit an association with control mothers.

MI error group										
Variants	Type	Location	Amino acid change	Base position	Genotype	Case (N = 700)	Control (N = 730)	Odds ratio	95% CI	P-value (<0.02)
rs62422269 A>G	5’ UTR variant	5’ Upstream sequence	N.A	118935183	AA	0.37	0.39	Reference		
					AG	0.48	0.45	1.124	0.895–1.410	0.325
					GG	0.15	0.16	0.996	0.728–1.362	1
rs114000233 T>G	5’ UTR variant	5’ Upstream sequence	N.A	118935167	TT	0.42	0.51	Reference		
					TG	0.46	0.45	1.238	0.997–1.538	0.053
					GG	0.12	0.04	**3.665**	**2.339–5.743**	<0.0001*
rs1885125 T>C	5’ UTR variant	(Promoter) ENSE00002068426	N.A	118934914	TT	0.57	0.6	Reference		
					TC	0.36	0.3	1.263	1.007–1.584	0.044
					CC	0.07	0.1	1.342	0.902–1.996	0.147
rs62422267 G>C	5’ UTR variant	(Promoter) ENSE00002068426	N.A	118935008	GG	0.42	0.44	Reference		
					GC	0.34	0.37	0.962	0.760–1.218	0.764
					CC	0.24	0.19	1.320	1.002–1.737	0.050
rs62422268 C>G	5’ UTR variant	(Promoter) ENSE00002068426	N.A	118935067	CC	0.36	0.41	Reference		
					CG	0.41	0.45	1.035	0.822–1.303	0.814
					GG	0.23	0.14	**1.873**	**1.388–2.527**	<0.0001*
rs72966896 T>C	5’ UTR variant	(Promoter) ENSE00002068426	N.A	118934917	TT	0.41	0.47	Reference		
					TC	0.32	0.3	1.222	0.958–1.559	0.107
					CC	0.27	0.23	1.345	1.036–1.744	0.029
rs62422266 T>C	5’ UTR variant	(Promoter) ENSE00002068426	N.A	118935000	TT	0.46	0.49	Reference		
					TC	0.36	0.39	0.983	0.784–1.233	0.908
					CC	0.18	0.12	**1.610**	**1.179–2.200**	0.003*
rs750913698 G>A	5’ UTR variant	(Promoter) ENSE00002046152	N.A	118931724	GG	0.35	0.31	Reference		
					GA	0.51	0.48	0.945	0.749–1.193	0.677
					**AA**	0.14	0.21	**0.594**	**0.435–0.810**	0.001*
rs188323243/MH979673T>G	Missense (Novel)	(Exon 2) ENSE00002046152	Valine-Glutamine	118931602	TT	0.81	0.54	Reference		
					TG	0.15	0.32	**0.312**	**0.239–0.406**	<0.0001*
					GG	0.04	0.14	**0.191**	**0.123–0.296**	<0.0001*
MK599406 G>T	Missense (Novel)	(Exon 2) ENSE00002046152	Glutamic acid- Aspartic acid	118931682	GG	0.74	0.69	Reference		
					GT	0.21	0.25	0.780	0.608–1.001	0.057
					TT	0.04	0.06	0.618	0.379–1.008	0.067
MK647974 G>A	Missense (Novel)	(Exon 2) ENSE00002046152	Glutamic acid- Lysine	118931669	GG	0.68	0.59	Reference		
					GA	0.26	0.31	**0.729**	**0.577–0.922**	0.009*
					AA	0.06	0.1	**0.521**	**0.349–0.778**	0.002*
MK647975 G>A	Synonymous (Novel)	(Exon 2) ENSE00002046152	Lysine	118931658	GG	0.48	0.5	Reference		
					GA	0.38	0.3	**1.319**	**1.046–1.664**	0.021*
					AA	0.14	0.2	0.729	0.542–0.980	0.037
MK647976 G>A	Missense (Novel)	(Exon 2) ENSE00002046152	Aspartic acid- Asparagine	118931621	GG	0.38	0.42	Reference		
					GA	0.5	0.52	1.063	0.854–1.324	0.615
					AA	0.12	0.06	**2.523**	**1.697–3.751**	<0.0001*
MK647977 G>A	Synonymous (Novel)	(Exon 2) ENSE00002046152	Glutamic acid	118931577	GG	0.43	0.49	Reference		
					GA	0.47	0.45	1.189	0.958–1.477	0.123
					AA	0.1	0.06	**1.936**	**1.285–2.916**	0.002*
MK647978 G>C	Missense (Novel)	(Exon 2) ENSE00002046152	Alanine-Proline	118931497	GG	0.39	0.4	Reference		
					GC	0.48	0.58	0.849	0.683–1.057	0.148
					CC	0.13	0.02	**6.489**	**3.667–11.482**	<0.0001*
MK647979 G>C	Missense (Novel)	(Exon 2) ENSE00002046152	Glutamine-Histidine	118931466	GG	0.61	0.22	Reference		
					GC	0.24	0.59	**0.147**	**0.114–0.190**	<0.0001*
					CC	0.15	0.19	**0.285**	**0.209–0.389**	<0.0001*
rs374755975 G>A	5’UTR variant	(Promoter of Exon 2) ENSE00002046152	N.A.	118931731	GG	0.7	0.65	Reference		
					GA	0.2	0.27	**0.689**	**0.536–0.885**	**0.004**
					AA	0.1	0.08	1.170	0.808–1.694	0.452
rs1259352607 T>G	Missense	(Exon 2) ENSE00002046152	Leucine-Arginine	118931701	TT	0.51	0.47	Reference		
					TG	0.39	0.45	0.797	0.641–0.992	0.045
					GG	0.1	0.08	1.160	0.794–1.693	0.501
rs1370486625 G>T	Intron/Splice acceptor variant	Intron 1–2	N.A.	118931739	GG	0.61	0.59	Reference		
					GT	0.36	0.4	0.871	0.702–1.080	0.228
					TT	0.03	0.01	**3.028**	**1.274–7.199**	0.011*
rs531682044 G>A	Missense	(Exon 2) ENSE00002046152	Arginine-Glutamine	118931503	GG	0.32	0.4	Reference		
					GA	0.46	0.4	**1.438**	**1.136–1.819**	0.003*
					AA	0.22	0.2	1.375	1.033–1.830	0.029
rs73521381 T>G	Synonymous variant	(Exon 2) ENSE00002046152	Serine	118931511	TT	0.38	0.71	Reference		
					TG	0.51	0.21	**4.553**	**3.580–5.789**	<0.0001*
					GG	0.11	0.08	**2.590**	**1.786–3.756**	<0.0001*
rs755141674 G>A	Missense	(Exon 2) ENSE00002046152	Glutamine-Histidine	118931442	GG	0.67	0.5	Reference		
					GA	0.31	0.4	**0.578**	**0.462–0.722**	<0.0001*
					AA	0.02	0.1	**0.153**	**0.086–0.272**	<0.0001*
rs770564988 G>A	Intron variant	Intron 1–2	N.A.	118931380	GG	0.79	0.48	Reference		
					GA	0.18	0.38	**0.287**	**0.224–0.369**	<0.0001*
					AA	0.03	0.14	**0.132**	**0.081–0.212**	<0.0001*
rs1267215855 T>C	Synonymous variant	(Exon 3) ENSE00001160217	Proline	118924111	TT	0.52	0.99	Reference		
					**TC**	0.32	0.01	**63.560**	**29.63–136.31**	<0.0001*
					**CC**	0.16	-	**446.60**	**27.66–209.6**	<0.0001*
rs140838152 A>G	Missense	(Exon 3) ENSE00001160217	Glutamic acid- Valine	118924109	AA	0.63	1	Reference		
					**AG**	0.31	**-**	**719.75**	**44.733–11581**	<0.0001*
					**GG**	0.06	**-**	**140.64**	**8.627–2292.6**	<0.0001*
rs1331061317 G>A	synonymous	(Exon 3) ENSE00001160217	Glutamic acid	118924108	GG	0.43	0.61	Reference		
					**GA**	0.41	0.32	**1.813**	**1.446–2.274**	<0.0001*
					**AA**	0.16	0.07	**3.247**	**2.260–4.663**	<0.0001*
rs576382724 A>C	Missense	(Exon 3) ENSE00001160217	Histidine-Proline	118924067	AA	0.36	0.55	Reference		
					**AC**	0.42	0.32	**2.004**	**1.588–2.530**	<0.0001*
					**CC**	0.22	0.13	**2.586**	**1.915–3.492**	<0.0001*
rs375494814 T>C	synonymous	(Exon 3) ENSE00001160217	Serine	118924057	TT	0.51	0.63	Reference		
					**TC**	0.38	0.28	**1.680**	**1.336–2.112**	<0.0001*
					CC	0.11	0.09	1.503	1.052–2.148	0.029
rs367896634 G>C	missense	(Exon 3) ENSE00001160217	Arginine-Glutamine	118924037	GG	0.46	1	Reference		
					**GC**	0.33	-	**1048.7**	**65.155–16881**	<0.0001*
					**CC**	0.21	-	**668.21**	**41.443–10774**	<0.0001*
rs1322432805 G>A	Missense	(Exon 3) ENSE00001160217	Lysine	118924021	GG	0.28	0.45	Reference		
					**GA**	0.52	0.42	**1.990**	**1.576–2.513**	<0.0001*
					**AA**	0.2	0.13	**2.474**	**1.805–3.389**	<0.0001*
rs1316687536 T>G	Missense	(Exon 3) ENSE00001160217	Valine- Alanine	118924019	TT	0.54	0.88	Reference		
					**TG**	0.38	0.12	**5.134**	**3.909–6.742**	<0.0001*
					**GG**	0.08	-	**191.82**	**11.810–3115.6**	<0.0001*
rs1486475303 C>A	Synonymous	(Exon 3) ENSE00001160217	Tyrosine	118923997	CC	0.43	1	Reference		
					**CA**	0.41	-	**1335.9**	**83.030–21495**	<0.0001*
					**AA**	0.16	-	**522.76**	**32.369–8442.7**	<0.0001*
rs1364710617 T>G	Missense	(Exon 3) ENSE00001160217	Cystine-Arginine	118923993	TT	0.55	0.92	Reference		
					**TG**	0.34	0.08	**7.162**	**5.236–9.797**	<0.0001*
					**GG**	0.11	-	**270.40**	**16.704–4377**	<0.0001*
rs754872940 A>C	Missense	(Exon 3) ENSE00001160217	Histidine-Arginine	118923977	AA	0.37	0.93	Reference		
					**AC**	0.49	0.07	**17.632**	**12.711–24.457**	<0.0001*
					**CC**	0.14	-	**515.84**	**31.9–8341.6**	<0.0001*
rs549531759 G>A	Missense	(Exon 3) ENSE00001160217	Glutamine-Histidine	118923946	GG	0.38	0.1	Reference		
					**GA**	0.36	-	**139.28**	**8.578–2261.3**	<0.0001*
					**AA**	0.26	-	**100.67**	**6.194–1636.1**	<0.0001*
rs764822140 C>A	Stop gained	(Exon 3) ENSE00001160217	Tyrosine	118923940	CC	0.41	0.53	Reference		
					CA	0.30	0.41	0.947	0.749–1.196	0.677
					**AA**	0.29	0.06	**6.221**	**4.340–8.917**	<0.0001*
rs573678849 G>A	Synonymous	(Exon 3) ENSE00001160217	Glutamic acid	118923904	GG	0.34	0.48	Reference		
					GA	0.36	0.41	1.243	0.983–1.572	0.072
					**AA**	0.3	0.11	**3.871**	**2.852–5.256**	<0.0001*
rs370725680 T>C	synonymous	(Exon 3) ENSE00001160217	Serine	118923889	TT	0.35	0.49	Reference		
					**TC**	0.51	0.43	**1.661**	**1.330–2.075**	<0.0001*
					**CC**	0.14	0.08	**2.469**	**1.717–3.550**	<0.0001*
rs7746114 C>T	Intron variant	Intron 3–4	N.A.	118923680	CC	0.35	0.38	Reference		
					CT	0.45	0.42	1.160	0.919–1.464	0.213
					TT	0.2	0.2	1.084	0.812–1.447	0.607
rs9374756 A>G	Intron variant	Intron 5–6	N.A.	118913523	AA	0.42	0.38	Reference		
					AG	0.49	0.44	1.010	0.808–1.264	0.955
					**GG**	0.09	0.18	**0.455**	**0.323–0.640**	<0.0001*
rs41292550 C>T	Intron variant	Intron 5–6	N.A.	118913534	CC	0.53	0.56	Reference		
					CT	0.37	0.32	1.220	0.974–1.529	0.085
					TT	0.1	0.12	0.887	0.628–1.252	0.539

Colour Code-

**Red**- For variants that were found to be risk factors based on significant P values and Odds ratios> 1.

**Green**- For variants that were found to be protective based on P values and Odds ratios<1.

* P-value obtained through Fisher’s exact test. P-value after Bonferroni’s Correction- ‘<0.02’ is considered as statistically significant. P-value is obtained as follows- 0.05/3 = 0.0166 or 0.02. ‘3’ is the number of genotypes. The 95% CI was calculated using the approximation of Woolf. Also, the P values, odds ratio, and the 95% CIs have been rounded to three decimal places to equilibrate the data.

**Table 3:** Distribution of MCM9 genotypes among the control mothers (N = 730) and mothers of children with Down syndrome (N = 125) who experienced meiosis II errors. This table shows MCM9 variants are not associated with Meiosis II errors.

MII Error group										
Variants	Type	Location	Amino acid change	Base position	Genotype	Case (N = 125)	Control (N = 730)	Odds ratio	95% CI	P-value (<0.02)
rs62422269 A>G	Intronic variant	5’ Upstream sequence	N.A	118935183	AA	0.44	0.39	Reference		
					AG	0.34	0.45	0.662	0.429–1.019	0.064
					GG	0.22	0.16	1.251	0.756–2.070	0.429
rs114000233 T>G	Intronic variant	5’ Upstream sequence	N.A	118935167	TT	0.51	0.51	Reference		
					TG	0.41	0.45	0.901	0.606–1.340	0.616
					GG	0.08	0.04	2.004	0.931–4.313	0.102
rs1885125 T>C	5’ UTR variant	(Promoter) ENSE00002068426	N.A	118934914	TT	0.56	0.6	Reference		
					TC	0.39	0.3	1.400	0.939–2.088	0.116
					CC	0.05	0.1	0.514	0.216–1.228	0.151
rs62422267 G>C	5’ UTR variant	(Promoter) ENSE00002068426	N.A	118935008	GG	0.4	0.44	Reference		
					GC	0.34	0.37	1.022	0.659–1.585	1
					CC	0.26	0.19	1.478	0.909–2.404	0.122
rs62422268 C>G	5’ UTR variant	(Promoter) ENSE00002068426	N.A	118935067	CC	0.5	0.41	Reference		
					CG	0.4	0.45	0.721	0.482–1.079	0.125
					GG	0.1	0.14	0.558	0.289–1.077	0.104
rs72966896 T>C	5’ UTR variant	(Promoter) ENSE00002068426	N.A	118934917	TT	0.48	0.47	Reference		
					TC	0.37	0.3	1.201	0.789–1.827	0.389
					CC	0.15	0.23	0.647	0.374–1.118	0.121
rs62422266 T>C	5’ UTR variant	(Promoter) ENSE00002068426	N.A	118935000	TT	0.5	0.49	Reference		
					TC	0.37	0.39	0.917	0.608–1.383	0.754
					CC	0.13	0.12	1.045	0.575–1.898	0.879
rs188323243 T>G	Missense (Novel)	(Promoter) ENSE00002046152	N.A	118931602	TT	0.39	0.31	Reference		
					TG	0.42	0.48	0.688	0.450–1.052	0.099
					GG	0.19	0.21	0.727	0.428–1.234	0.295
MK599406 G>T	Missense (Novel)	(Exon 2) ENSE00002046152	Valine-Glutamine	118931682	GG	0.52	0.54	Reference		
					GT	0.37	0.32	1.192	0.79–1.797	0.399
					TT	0.11	0.14	0.832	0.449–1.542	0.652
MK647974 G>A	Missense (Novel)	(Exon 2) ENSE00002046152	Glutamic acid- Aspartic acid	118931669	GG	0.62	0.69	Reference		
					GA	0.30	0.25	1.304	0.851–1.997	0.259
					AA	0.08	0.06	1.466	0.708–3.032	0.304
MK647975 G>A	Synonymous (Novel)	(Exon 2) ENSE00002046152	Glutamic acid- Lysine	118931658	GG	0.51	0.59	Reference		
					GA	0.4	0.31	1.302	0.867–1.955	0.208
					AA	0.09	0.1	0.984	0.496–1.952	1
MK647976 G>A	Missense (Novel)	(Exon 2) ENSE00002046152	Lysine	118931621	GG	0.47	0.5	Reference		
					GA	0.41	0.3	1.441	0.956–2.172	0.088
					AA	0.12	0.2	0.636	0.349–1.156	0.164
MK647977 G>A	Synonymous (Novel)	(Exon 2) ENSE00002046152	Aspartic acid- Asparagine	118931577	GG	0.4	0.42	Reference		
					GA	0.5	0.52	1.371	0.919–2.046	0.123
					AA	0.1	0.06	2.165	1.088–4.305	0.05
MK647978 G>C	Missense (Novel)	(Exon 2) ENSE00002046152	Glutamic acid	118931497	GG	0.47	0.49	Reference		
					GC	0.42	0.45	0.959	0.642–1.433	0.919
					CC	0.11	0.06	1.976	1.018–3.834	0.05
MK647979 G>C	Missense (Novel)	(Exon 2) ENSE00002046152	Alanine-Proline	118931466	GG	0.45	0.4	Reference		
					GC	0.54	0.58	0.821	0.559–1.205	0.324
					CC	0.01	0.02	0.348	0.045–2.686	0.484
rs374755975 G>A	5’ UTR variant	(Exon 2) ENSE00002046152	Glutamine-Histidine	118931731	GG	0.2	0.22	Reference		
					GA	0.57	0.59	1.063	0.651–1.737	0.902
					AA	0.23	0.19	1.344	0.751–2.403	0.375
rs1259352607 T>G	Missense	(Exon 2) ENSE00002046152	N.A.	118931701	TT	0.62	0.65	Reference		
					TG	0.3	0.27	1.144	0.748–1.750	0.581
					GG	0.08	0.08	1.050	0.515–2.141	0.855
rs1370486625 G>T	Splice acceptor variant	(Exon 2) ENSE00002046152	Leucine-Arginine	118931739	GG	0.51	0.47	Reference		
					GT	0.35	0.45	0.701	0.463–1.061	0.096
					TT	0.14	0.08	1.663	0.919–3.008	0.097
rs531682044 G>A	Missense	Intron 1–2	N.A.	118931503	GG	0.65	0.59	Reference		
					GA	0.34	0.4	0.756	0.507–1.128	0.196
					AA	0.01	0.01	0.751	0.091–6.188	1
rs73521381 T>G	Synonymous variant	(Exon 2) ENSE00002046152	Arginine-Glutamine	118931511	TT	0.5	0.4	Reference		
					TG	0.35	0.4	0.698	0.459–1.061	0.094
					GG	0.14	0.2	0.571	0.326–1.001	0.051
rs750913698 G>A	5’ UTR variant	(Exon 2) ENSE00002046152	Serine	118931724	GG	0.68	0.71	Reference		
					GA	0.21	0.21	1.038	0.645–1.668	0.903
					AA	0.11	0.08	1.474	0.787–2.760	0.219
rs755141674 G>A	Missense	(Exon 2) ENSE00002046152	Glutamine-Histidine	118931442	GG	0.6	0.5	Reference		
					GA	0.34	0.4	0.7	0.465–1.054	0.104
					AA	0.06	0.1	0.533	0.246–1.155	0.134
rs770564988 G>A	Intron variant	Intron 1–2	N.A.	118931380	GG	0.5	0.48	Reference		
					GA	0.4	0.38	0.817	0.541–1.234	0.352
					AA	0.1	0.14	0.656	0.348–1.236	0.239
rs1267215855 T>C	Synonymous variant	(Exon 3) ENSE00001160217	Proline	118924111	TT	0.99	0.99	Reference		
					TC	0.01	0.01	0.833	0.102–6.832	1
					CC	-	-	-	-	-
rs140838152 A>G	Missense	(Exon 3) ENSE00001160217	Glutamic acid- Valine	118924109	AA	1	1	Reference		
					AG	-	**-**	-	-	-
					GG	-	**-**	-	-	-
rs1331061317 G>A	Synonymous	(Exon 3) ENSE00001160217	Glutamic acid	118924108	GG	0.7	0.61	Reference		
					GA	0.24	0.32	0.648	0.416–1.010	0.057
					AA	0.06	0.07	0.6941	0.305–1.580	0.455
rs576382724 A>C	Missense	(Exon 3) ENSE00001160217	Histidine-Proline	118924067	AA	0.53	0.55	Reference		
					AC	0.30	0.32	0.989	0.643–1.521	1
					CC	0.17	0.13	1.346	0.785–2.310	0.308
rs375494814 T>C	synonymous	(Exon 3) ENSE00001160217	Serine	118924057	TT	0.61	0.63	Reference		
					TC	0.3	0.28	1.127	0.739–1.721	0.585
					CC	0.09	0.09	1.009	0.509–1997	1
rs367896634 G>C	Missense	(Exon 3) ENSE00001160217	Arginine-Glutamine	118924037	GG	1	1	Reference		
					GC	-	-	-	-	-
					CC	-	-	-	-	-
rs1322432805 G>A	Missense	(Exon 3) ENSE00001160217	Lysine	118924021	GG	0.48	0.45	Reference		
					GA	0.42	0.42	0.947	0.634–1.414	0.838
					AA	0.1	0.13	0.693	0.358–1.341	0.352
rs1316687536 T>G	Missense	(Exon 3) ENSE00001160217	Valine- Alanine	118924019	TT	0.88	0.88	Reference		
					TG	0.12	0.12	0.995	0.555–1.784	1
					GG	-	-			
rs1486475303 C>A	Synonymous	(Exon 3) ENSE00001160217	Tyrosine	118923997	CC	1	1	Reference		
					CA	-	-	-	-	-
					AA	-	-	-	-	-
rs1364710617 T>G	Missense	(Exon 3) ENSE00001160217	Cystine-Arginine	118923993	TT	0.84	0.92	Reference		
					TG	0.09	0.08	1.324	0.688–2.549	0.370
					**GG**	**0.07**	-	**108.36**	**6.204–1892.7**	<0.0001*
rs754872940 A>C	Missense	(Exon 3) ENSE00001160217	Histidine-Arginine	118923977	AA	0.9	0.93	Reference		
					AC	0.1	0.07	1.414	0.731–2.735	0.352
					CC	-	-	-	-	-
rs549531759 G>A	Missense	(Exon 3) ENSE00001160217	Glutamine-Histidine	118923946	GG	1	0.1	Reference		
					GA	-	-	-	-	-
					AA	-	-	-	-	-
rs764822140 C>A	Stop gained	(Exon 3) ENSE00001160217	Tyrosine	118923940	CC	0.5	0.53	Reference		
					CA	0.4	0.41	1.027	0.688–1.534	0.919
					AA	0.1	0.06	1.675	0.839–3.346	0.161
rs573678849 G>A	Synonymous	(Exon 3) ENSE00001160217	Glutamic acid	118923904	GG	0.5	0.48	Reference		
					GA	0.42	0.41	0.969	0.651–1.443	0.919
					AA	0.08	0.11	0.696	0.342–1.417	0.409
rs370725680 T>C	synonymous	(Exon 3) ENSE00001160217	Serine	118923889	TT	0.47	0.49	Reference		
					TC	0.41	0.43	0.986	0.658–1.477	1
					CC	0.12	0.08	1.569	0.835–2.950	0.159
rs7746114 C>T	Intron variant	Intron 3–4	N.A.	118923680	CC	0.36	0.38	Reference		
					CT	0.44	0.42	1.103	0.720–1.689	0.666
					TT	0.2	0.2	1.054	0.621–1.788	0.892
rs9374756 A>G	Intron variant	Intron 5–6	N.A.	118913523	AA	0.49	0.38	Reference		
					AG	0.37	0.44	1.6	1.035–2.474	0.04
					GG	0.14	0.18	0.626	0.356–1.102	0.112
rs41292550 C>T	Intron variant	Intron 5–6	N.A.	118913534	CC	0.52	0.56	Reference		
					CT	0.36	0.32	1.210	0.801–1.828	0.393
					TT	0.12	0.12	1.085	0.591–1.991	0.754

Colour Code-

**Red**- Variants found to be risk factors based on significant P values and Odds ratios> 1.

**Green**- Variants found to be protective also based on P values and Odds ratios<1.

* P-value after Bonferroni’s Correction- ‘<0.02’ is considered as statistically significant. The P-value was obtained as follows- 0.05/3 = 0.0166 or 0.02. ‘3’ is the number of genotypes. The 95% CI was calculated using an approximation of Woolf. Also, the P values, odds ratios, and the 95% CIs were rounded to three decimal places to equilibrate the data.

All variants were found in the upstream UTR sequence, exon 2 and exon 3 of the MCM9 gene. No variants were found in the remaining exons or in the conserved ATP-binding domains (i.e., the multiple ATP binding sites spanning from 314V to 567R residue that correspond to exon 6, 7, 8, 9, and 10 of the reading frame).

#### Novel polymorphic sites and novel alleles

**Fig 1:**
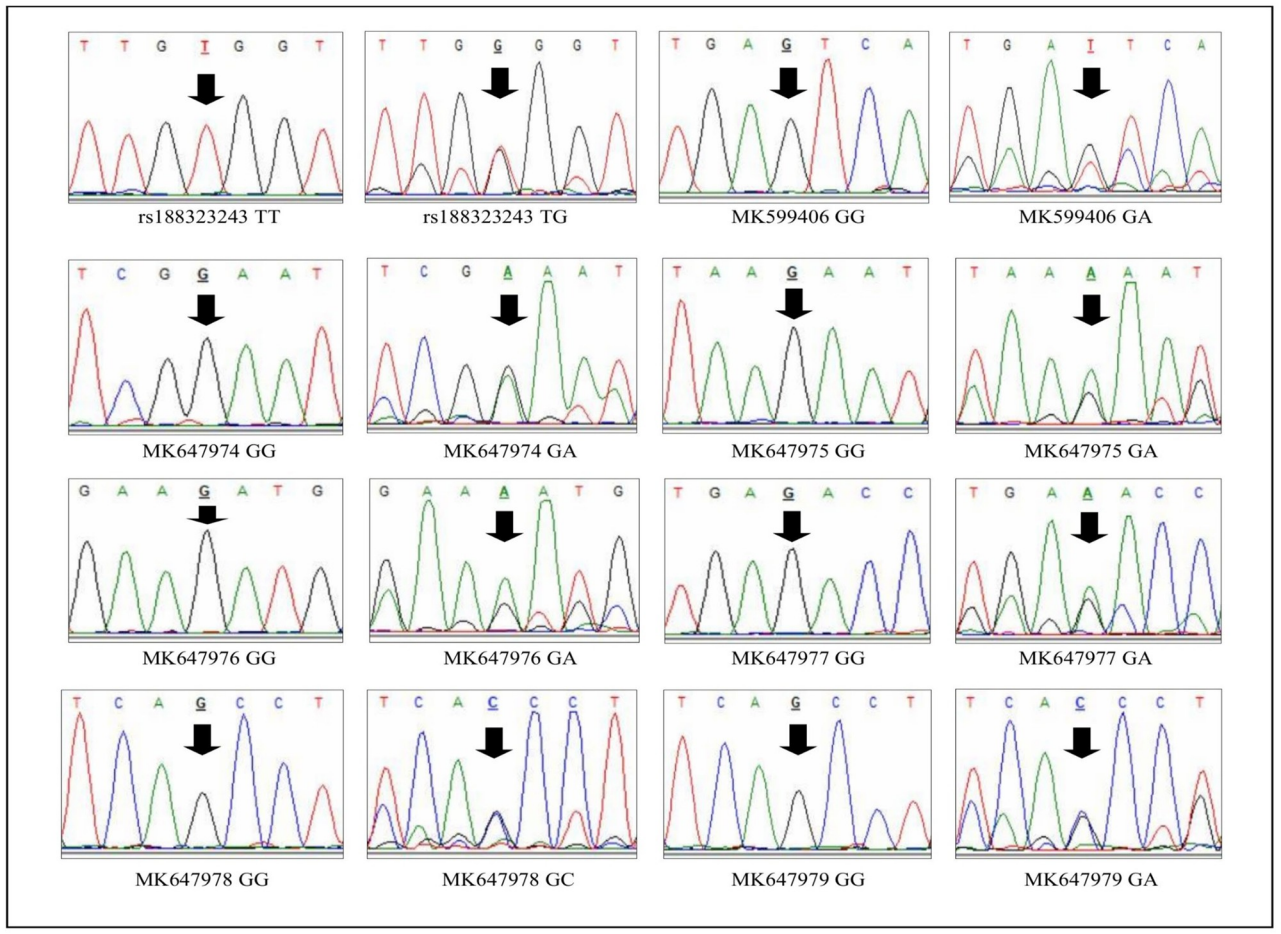
Chromatograms show wild type homozygous alleles and mutant heterozygous novel alleles in the control (mothers with euploid child) and case (mothers of DS child) samples found within exon 2 of the MCM9 gene.

We found six novel polymorphic sites (Fig 1) within exon 2 that were unique in the Bengali population. We submitted these novel variants to NCBI Gen-Bank for validation and received accession numbers MK599406 G>T (neutral allele), MK647974G>A (protective allele), MK647975G>A (risk allele), MK647976G>A (risk allele), MK647977G>A (risk allele), and MK647979G>C (protective allele). Each of these variants cause amino acid replacements. The anticipated changes in the MCM9 amino acid sequences are MK599406 G>T glutamic acid to aspartic acid, MK647974 G>A glutamic acid to lysine, MK647975 G>A synonymous lysine, MK647976 G>A aspartic acid to asparagine, MK647977 G>A synonymous glutamic acid, MK647979 G>C glutamine to histidine. Also, we found two novel alleles for the polymorphic sites rs188323243 and rs771165705 within exon 2. For rs188323243 (protective allele) the reference (NCBI GDB) major and minor alleles were ‘T’ and ‘A’ respectively, but we found a minor allele ‘G’ in our population. This change causes an amino acid replacement of valine to glutamic acid. We received an accession number for this new allele as MH979673T>G (protective allele). For the SNP rs771165705, the reference replacement was C>T that caused an amino acid change from leucine to serine, but we found the replacement G>C (risk allele), which causes an anticipatory missense change from alanine to proline in the MCM9 protein. The accession number for this new variant is MK647978 G>C (Table 2).

#### Polymorphisms associated with MI error

This section describes the association of MCM9 risk variants with MI errors. We found twenty-five variants out of forty-one as ‘risk’ for MI errors and these variants altogether constituted thirty-eight maternal ‘risk genotypes’. We observed a strong association between two promoter (ENSE00002068426) variants, namely rs62422268 (GG) and rs62422266 (CC) with MI error. The estimated odds for the risk of NDJ among risk genotype-MI mothers was 1.8 (rs62422268) and 1.6 (rs62422266) times that of controls (Table 2). Also, the homozygous ‘GG’ genotype of variant rs114000233, which occurs in the 5´ UTR region, increases the odds of NDJ in favour of case mothers by 3.7 (approx.) times.

We detected twelve SNPs in exon 2 (ENSE00002046152) (Table 2). Out of these 6 were from the ‘risk’ category, and the rest were either ‘neutral’ or ‘protective’. The heterozygous ‘GA’ genotype of the novel variant MK647975, the homozygous ‘AA’ genotype of the novel variant MK647976, the homozygous ‘AA’ genotype of the novel variant MK647977, the homozygous ‘CC’ of the novel variant MK647978, the heterozygous ‘GA’ of rs531682044, and both heterozygous ‘TG’ and homozygous ‘GG’ of the variant rs73521381 exhibited elevated odds infavor of MI-NDJ errors (Table 2). Similarly, the homozygous ‘TT’ genotype of intronic variant rs1370486625 exhibited odds in favor of MI error by 3.028 times.

We observed fifteen SNPs in exon 3 (Table 2). Interestingly, four variants rs140838152, rs367896634, rs1486475303, and rs549531759 (minor allele), were found present only in the case samples, but not in controls. Both heterozygous and homozygous genotypes of the above-mentioned variants exhibited odds in favor of MI error among case women than controls. Additionally, for rs1267215855, rs1316687536, rs1364710617, and rs754872940, we observed homozygous genotypes for minor alleles only in case samples and found the odds in favour of MI error. The heterozygous genotypes of these four polymorphic sites were found in control women and again their estimated odds were in favor of MI errors (Table 2).

Similarly, the heterozygous and homozygous genotypes of the exon 3 variants rs1331061317 (GA and AA), rs576382724 (AC and CC), rs1322432805 (GA and AA) and rs370725680 (TC and CC) (Table 2) exhibited elevated odds in favor of MI errors than controls. The remaining three variants, (i.e., the heterozygous ‘TC’ genotype of rs375494814, homozygous ‘AA’ genotype of rs764822140, and homozygous ‘AA’ genotype of rs573678849) also demonstrated odds in favor of MI errors (Table 2).

#### Polymorphisms protective against MI error

Our analyses revealed eight variants of MCM9 in which the minor allele exhibited a negative association within the MI error group (Table 2); in other words, these variants were more likely to occur among the control women. The homozygous ‘AA’ genotype of rs750913698 in the 5´UTR region exhibited decreased odds in favor of MI errors. The heterozygous ‘GA’ genotype of rs374755975 in the promoter of exon 2, the heterozygous and homozygous genotypes of rs188323243/MH979673 (TG and GG), MK647974 (GA and GG), MK647979 (GC and CC) and rs755141674 (GA and AA) also showed protective association. Similarly, the presence of both heterozygous ‘GA’and homozygous ‘AA’ genotypes of the intronic variant rs770564988, and the homozygous ‘GG’ genotype of the rs9374756 also showed reduced odds for MI errors among case mothers when compared to controls (Table 2). We considered these polymorphisms as ‘protective’ against maternal MI errors.

#### MI neutral polymorphisms

Eight polymorphic sites exhibited no significant association with either the MI error group or with the control group (Table 2). We considered them as ‘neutral’ sites. These included the 5´ UTR upstream site variant rs62422269 A>G, the promoter site variants rs1885125 T>C, rs62422267 G>C, rs72966896 T>C, and MK599406 G>T (which codes for the novel minor ‘T’ allele among Bengali women and causes a missense replacement), the exon 2 variant rs125952607 T>G (causes missense amino acid change), and the intronic variants (located between exon 3 and 4) rs7746114 C>A, and rs412925550 C>T (located between exon 5 and 6).

#### Polymorphisms associated with MII error

We also performed an association study for the MII error group. Previously [2] it was observed that recombination anomalies increase the risk of MII errors in a different way than it does for MI errors. Intuitively, the polymorphisms of recombination regulator genes that exhibit association with MI NDJ may show different risk association with MII NDJ. The genotype distribution of MII women and the association of polymorphic sites are represented in Table 3. Surprisingly, we found only one variant, rs1364710617 T>G as a significant risk factor for MII errors. It was present only among MII error women, and not in controls. The estimated odds of the homozygous ‘GG’ genotype in favor of MII error is 108.36 times that of controls (Table 3). This nucleotide change causes cystine to arginine replacement in the MCM9 protein (Table 3). In addition, many of the polymorphic sites that were identified as significant risk factors for MI errors also showed a higher frequency of risk genotypes among MII women, though the associations were not statistically significant after Bonferroni correction.

#### Model wise distribution (dominant and recessive models)

In addition to individual genotype analysis, we were interested to explore the dominant and recessive models for MI NDJ risk. This model-based analysis enabled us to understand the “quantum of risk” imparted by the individual genotype in complex genetic risk scenarios. We designated dominant and recessive models for each polymorphic site for the MI women presented in Table 4. For the dominant models, the major homozygous genotypes served as the reference while for the recessive models, the sum of frequencies for the major homozygous genotypes and heterozygous genotypes served as the reference. The dominant recessive models of 21 polymorphic sites exhibited an association with MI errors. The variants rs114000233 T>G, rs62422268 C>G, rs62422266 T>C, MK647976 G>A, MK647977 G>A, MK647978 G>C, 1370486625 G>T, rs531682044 G>A, rs73521381 T>G, rs1267215855 T>C, rs140838152 A>G, rs1331061317 G>A, rs576382724 A>C, rs375494814 T>C, rs367896634 G>C, rs1322432805 G>A, rs1316687536 T>G, rs1486475303 C>A, rs1364710617 T>G, rs754872940 A>C, rs549531759 G>A, rs764822140 C>A, rs573678849 G>A and rs370725680 T>C, which previously exhibited an association with MI errors as individual genotypes (Table 1), also showed an association in their respective dominant and recessive models (Table 4).

**Table 4:** Model wise distribution of polymorphic variants showing the combined risk of genotypes among mothers’ who experienced MI error.

Variants	Model		Odds ratio	95% CI	P-value (<0.03)
**rs62422269 A>G**	Dominant	AA vs. AG+GG	1.09	0.881–1.350	0.446
	Recessive	AA+AG vs. GG	0.934	0.701–1.245	0.661
**rs114000233 T>G**	**Dominant**	**TT vs. TG+GG**	**1.435**	**1.165–1.768**	0.001*
	**Recessive**	**TT+TG vs. GG**	**3.296**	**2.132–5.097**	<0.0001*
**rs1885125 T>C**	Dominant	TT vs. TC+CC	1.132	0.917–1.397	0.259
	Recessive	TT+TC vs. CC	0.677	0.464–0.989	0.047
**rs62422267 G>C**	Dominant	GG vs. GC+CC	1.084	0.879–1.336	0.455
	Recessive	GG+GC vs. CC	1.343	1.042–1.730	0.024
**rs62422268 C>G**	Dominant	CC vs. CG+GG	1.233	0.996–1.527	0.057
	**Recessive**	**CC+CG vs. GG**	**1.839**	**1.399–2.418**	<0.0001*
**rs72966896 T>C**	Dominant	TT vs. TC+CC	1.275	1.034–1.572	0.025
	Recessive	TT+TC vs. CC	1.237	0.973–1.573	0.087
**rs62422266 T>C**	Dominant	TT vs. TC+CC	1.130	0.918–1.391	0.266
	**Recessive**	**TT+TC vs. CC**	1.601	1.193–2.150	0.002*
**rs750913698 G>A**	Dominant	GG vs. GA+AA	1.109	0.894–1.376	0.349
	**Recessive**	**GG+GA vs. AA**	**0.614**	**0.465–0.811**	0.001*
**rs188323243 T>G**	**Dominant**	**TT vs. TG+GG**	**0.275**	**0.217–0.349**	<0.0001*
	**Recessive**	**TT+TG vs. GG**	**0.257**	**0.167–0.395**	<0.0001*
**MK599406 G>T**	**Dominant**	**GG vs. GT+TT**	**0.749**	**0.593–0.944**	0.016*
	Recessive	GG+GT vs. TT	0.657	0.404–1.067	0.092
**MK647974 G>A**	**Dominant**	**GG vs. GA+AA**	**0.678**	**0.546–0.843**	0.0004*
	**Recessive**	**GG+GA vs. AA**	**0.573**	**0.387–0.853**	0.006*
**MK647975 G>A**	Dominant	GG vs. GA+AA	1.083	0.880–1.333	0.459
	**Recessive**	**GG+GA vs. AA**	**0.651**	**0.492–0.861**	0.003*
**MK647976 G>A**	Dominant	GG vs. GA+AA	1.211	0.981–1.497	0.076
	**Recessive**	**GG+GA vs. AA**	**2.438**	**1.672–3.554**	<0.0001*
**MK647977 G>A**	Dominant	GG vs. GA+AA	1.276	1.036–1.571	0.023
	**Recessive**	**GG+GA vs. AA**	**1.775**	**1.196–2.635**	0.004*
**MK647978 G>C**	Dominant	GG vs. GC+CC	1.043	0.843–1.289	0.705
	**Recessive**	**GG+GC vs. CC**	**7.123**	**4.081–12.431**	<0.0001*
**MK647979 G>C**	**Dominant**	**GG vs. GC+CC**	**0.181**	**0.144–0.228**	<0.0001*
	Recessive	GG+GC vs. CC	0.750	0.568–0.991	0.049
**rs374755975 G>A**	Dominant	GG vs. GA+AA	0.798	0.639–0.997	0.048
	Recessive	GG+GA vs. AA	1.287	0.894–1.854	0.195
**rs1259352607 T>G**	Dominant	TT vs. TG+GG	0.852	0.692–1.048	0.139
	Recessive	TT+TG vs. GG	1.287	0.894–1.854	0.195
**rs1370486625 G>T**	Dominant	GG vs. GT+TT	0.922	0.746–1.139	0.483
	**Recessive**	**GG+GT vs. TT**	**3.194**	**1.349–7.564**	0.007*
**rs531682044 G>A**	**Dominant**	**GG vs. GA+AA**	**1.417**	**1.140–1.760**	0.002*
	Recessive	GG+GA vs. AA	1.128	0.875–1.456	0.364
**rs73521381 T>G**	**Dominant**	**TT vs. TG+GG**	**4.013**	**3.217–5.007**	<0.0001*
	Recessive	TT+TG vs. GG	1.432	1.001–2.048	0.058
**rs755141674 G>A**	**Dominant**	**GG vs. GA+AA**	**0.697**	**0.569–0.854**	<0.001*
	**Recessive**	**GG+GA vs. AA**	**0.189**	**0.107–0.333**	<0.0001*
**rs770564988 G>A**	**Dominant**	**GG vs. GA+AA**	**0.245**	**0.194–0.309**	<0.0001*
	**Recessive**	**GG+GA vs. AA**	**0.190**	**0.118–0.308**	<0.0001*
**rs1267215855 T>C**	**Dominant**	**TT vs. TC+CC**	**95.341**	**44.624–203.7**	<0.0001*
	**Recessive**	**TT+TC vs. CC**	**279.29**	**17.313–4505.4**	<0.0001*
**rs140838152 A>G**	**Dominant**	**AA vs. AG+GG**	**858.73**	**53.398–13810**	<0.0001*
	**Recessive**	**AA+AG vs. GG**	**94.294**	**5.787–1536.3**	<0.0001*
**rs1331061317 G>A**	**Dominant**	**GG vs. GA+AA**	**2.070**	**1.676–2.556**	<0.0001*
	**Recessive**	**GG+GA vs. AA**	**2.536**	**1.789–3.595**	**<0.0001**
**rs576382724 A>C**	**Dominant**	**AA vs. AC+CC**	**2.172**	**1.757–2.686**	<0.0001*
	**Recessive**	**AA+AC vs. CC**	**1.908**	**1.441–2.527**	<0.0001*
**rs375494814 T>C**	**Dominant**	**TT vs. TC+CC**	**1.637**	**1.325–2.022**	<0.0001*
	Recessive	TT+TC vs. CC	1.243	0.879–1.758	0.219
**rs367896634 G>C**	**Dominant**	**GG vs. GC+CC**	1714.7	106.65–27568	<0.0001*
	**Recessive**	**GG+GC vs. CC**	569.35	35.323–9177	<0.0001*
**rs1322432805 G>A**	**Dominant**	**GG vs. GA+AA**	2.104	1.689–2.623	<0.0001*
	**Recessive**	**GG+GA vs. AA**	1.674	1.260–2.224	0.0004*
**rs1316687536 T>G**	**Dominant**	**TT vs. TG+GG**	6.215	4.754–8.124	<0.0001*
	**Recessive**	**TT+TG vs. GG**	128.08	7.892–2078.5	<0.0001
**rs1486475303 C>A**	**Dominant**	**CC vs. CA+AA**	1935.9	120.4–31126	<0.0001*
	**Recessive**	**CC+CA vs. AA**	**279.29**	**17.313–4505.4**	<0.0001*
**rs1364710617 T>G**	**Dominant**	**TT vs. TG+GG**	**9.480**	**6.975–12.884**	<0.0001*
	**Recessive**	**TT+TG vs. GG**	**181.6**	**11.227–2937.4**	<0.0001*
**rs754872940 A>C**	**Dominant**	**AA vs. AC+CC**	**22.669**	**16.406–31.324**	<0.0001*
	**Recessive**	**AA+AC vs. CC**	**238.85**	**14.794–3856.3**	<0.0001*
**rs549531759 G>A**	**Dominant**	**GG vs. GA+AA**	**239.67**	**14.778–3886.8**	<0.0001*
	**Recessive**	**GG+GA vs. AA**	**61.743**	**3.803–1002.5**	<0.0001*
**rs764822140 C>A**	**Dominant**	**CC vs. CA+AA**	**1.624**	**1.317–2.002**	<0.0001*
	**Recessive**	**CC+CA vs. AA**	**6.368**	**4.506–8.999**	<0.0001*
**rs573678849 G>A**	**Dominant**	**GG vs. GA+AA**	**1.798**	**1.452–2.226**	<0.0001*
	**Recessive**	**GG+GA vs. AA**	**3.482**	**2.624–4.621**	<0.0001*
**rs370725680 T>C**	**Dominant**	**TT vs. TC+CC**	**1.787**	**1.445–2.211**	<0.0001*
	**Recessive**	**TT+TC vs. CC**	**1.886**	**1.339–2.658**	0.0003*
**rs7746114 C>T**	Dominant	CC vs. CT+TT	1.136	0.915–1.409	0.249
	Recessive	CC+CT vs. TT	1	0.772–1.296	1
**rs9374756 A>G**	Dominant	AA vs. AG+GG	0.849	0.687–1.050	0.131
	**Recessive**	**AA+AG vs. GG**	**0.452**	**0.328–0.623**	<0.0001*
**rs41292550 C>T**	Dominant	CC vs. CT+TT	1.130	0.917–1.392	0.2647
	**Recessive**	**CC+CT vs. TT**	**0.522**	**0.372–0.733**	0.0002*

Colour Code-

**Red**- Variants found to be risk factors based on significant P values and Odds ratios>1.

**Green**- Variants found to protective, also based on P values and Odds ratios<1.

P-value after Bonferroni’s Correction- ‘<0.03’ is considered as statistically significant. The P-value wasobtained as follows- 0.05/2 = 0.025 or 0.03. ‘2’ is the number of combinations of variants, e.g., for dominant and recessive models. The 95% CI was calculated using the approximation of Woolf. Also, the P values, odds ratios, and the 95% CIs were rounded off up to three decimal places to equilibrate the data.

In contrast to the above-mentioned risk models, many polymorphic sites and their respective genotypic combinations were found to be inversely associated with MI errors and were considered as ‘protective’. The polymorphic variants rs750913698 G>A, rs188323243, (for which we found the novel allele MH979673 T>G,) MK599406 G>T, MK647974 G>A, MK647979 G>C, rs755141674 G>A rs770564988 G>A, and rs9374756 A>G, all exhibited protective association in both the dominant and recessive models, respectively. On contrary, the variant rs41292550 C>T, which previously exhibited a neutral association with MI errors in our case-control genotype analysis, showed a protective association in its recessive model (CC+CT vs.TT) (Table 4).

### Linkage disequilibrium analyses

We were interested to see whether the variants that we found in the case samples were in linkage disequilibrium [LD] and if they formed any susceptible haplotypes that predispose women to an increased risk for Ch21 segregation errors. To do this, we analyzed all twenty-five risk variants that exhibited an association with MI errors using the “Haploview” program. Out of these twenty-five variants, four possible haplotypes consisting of two risk variants, MK647977 G>A (exon 2) and rs114000233 T>G (promoter), were predicted (Table 5). Out of these four possible haplotypes, three revealed significant associations with MI errors: G-T (P = 1.899E^-5^), G-G (P = 0.0436), and A-G (P = 2.8548E^-6^) respectively. However, the estimated ‘R^2^ value’ for these four haplotypes was ‘0.07’ which suggests an weak linkage disequilibrium (LD). Therefore, it can be said the above mentioned genotypes constituted with these two variants are associated with MI errors in oocytes, but the loci are non-linked.

**Table 5:** Haplotype analysis revealed three polymorphic sites that constituted four haplotypes significantly associated with MI errors.

Alleles	Haplotype	Case ratio	Control ratio	Chi-square (χ^2^) Value	*P value
GT	0.492	0.533	0.453	18.297	**1.8899E**^-5^*
AT	0.200	0.202	0.197	0.112	0.7377
GG	0.198	0.182	0.212	4.072	0.0436*
AG	0.111	0.083	0.138	21.912	**2.8548E**^-6^*

* P-value obtained by using Chi-square (χ^2^) test.

α = 0.05

### The relationship between variants in MCM9 and the amount of recombination on Ch21q

#### MI error group

To estimate the effect of MCM9 variants on the amount of detectable recombination on the nondisjoined Ch21q as a function of maternal age, we stratified women initially into three genotype categories, namely women with ‘MCM9 neutral genotypes’, women with ‘MCM9 protective genotypes’ and women with ‘MCM9 risk genotypes’. Women were classified in the ‘protective genotypes’ group if they carried at least one protective variant and no risk variants. The ‘risk genotypes’ group was constituted similarly, with the case women carrying at least one risk variant and no protective variant. The small number of women who carried both risk and protective variants were classified as ‘neutral genotypes’ group. These three categories were further stratified by the maternal age at conception of the DS fetus as young (<28 years), middle aged (28years– 34 years), and older women (>34 years). Tables 6 and 7 shows the distributions of the number of observed recombination events (0, 1, and ≥2) in MI and MII cases, respectively, as a function of MCM9 genotype and maternal age at conception. “0” means no recombination events and is considered a risk factor for NDJ, “1” stands for normal single recombinant event and “≥2” stands for double or more recombinant events. We scored 85% of meiosis without any detectable recombination events among the MI young women with ‘MCM9 risk genotypes’, in contrast to 60% and 57% from the same age category of mothers having ‘neutral’ and ‘protective’ genotypes, respectively. This trend was similar for women from the middle and old-age categories. We scored 72% non-recombinant MI errors among the middle age ‘MCM9 risk genotypes’ mothers while the estimates for middle-aged mothers having ‘neutral’ and ‘protective’ genotypes were 53% and 51% respectively (Table 6). For the old age group, the scores for non-recombinant MI errors among the ‘MCM9 neutral’, ‘MCM9 protective’ and ‘MCM9 risk’ genotype groups were 35%, 37%, 67%, respectively, this implies that the chance of absence of recombination in women having the ‘risk genotypes’ is nearly twice the frequency observed among the women having either ‘neutral’ or ‘protective’ genotypes. We also observed a drop in the frequency of detectable single recombinant events across age groups within the MCM9 risk genotype group. The frequencies of single recombinant events in the young age group among the MCM9 neutral, protective and risk groups were 30%, 40% and 12%, respectively. Consistent with this trend, the frequencies of single recombination events in the middle-aged group having ‘neutral’, ‘protective’, and ‘risk’ genotypes were 41%, 38%, and 18%, respectively. Similarly, the old age group also exhibited a reduced frequency of single recombination events on Ch21q with the frequencies 57%, 60%, and 20% for the ‘neutral’, ‘protective’, and ‘risk’ genotype groups, respectively. We then compared the distribution of the amount of detectable recombination among the age categories from the three genotype groups in a pair-wise manner by Chi-squared test (Table 6). A significant difference was detected after comparing the ‘MCM9 neutral genotype’ and ‘MCM9 risk genotype’ groups (P = 0.0003 for young, P = 0.002 for middle and P<0.0001 for old). A significant difference was also detected after comparing the ‘MCM9 protective genotype’ with the ‘MCM9 risk genotype’ groups (P<0.0001 for young, P = 0.005 for middle and P<0.0001 for old), but not when comparing the ‘MCM9 neutral genotype’ and ‘MCM9 protective genotype’ groups.

**Table 6:** Frequency distribution of observed recombinants among MI errors stratified by maternal genotypes and maternal age group.

Genotype category of MI error group mothers	Age category	*N	Number of observed recombination			Chi-square value and P-value
			0	1	≥2	
MCM9 Neutral genotype	Young (<29 yrs.)	92	0.6	0.3	0.1	**Young (<29 yrs)-** **Neutral vs. Protective χ2 = 5.27, P = 0.07** **Neutral vs. Risk χ2 = 15.79, P = 0.0003** **Protective vs. Risk χ2 = 20.59, P<0.0001**
	Middle (29–34 yrs.)	81	0.53	0.41	0.06	
	Old (>34 yrs.)	63	0.35	0.57	0.07	
MCM9 Protective genotype	Young (<29 yrs.)	72	0.57	0.4	0.03	**Middle (29–34 yrs)-** **Neutral vs. Protective χ2 = 1.62, P = 0.4** **Neutral vs. Risk χ2 = 12.85,P = 0.002** **Protective vs. Risk χ2 = 10.78, P = 0.005**
	Middle (29–34 yrs.)	54	0.51	0.38	0.11	
	Old (>34 yrs.)	40	0.37	0.6	0.03	
MCM9-Risk genotype	Young (<29 yrs.)	144	0.85	0.12	0.03	**Old (>34 yrs)-** **Neutral vs. Protective χ2 = 2.41, P = 0.3** **Neutral vs. Risk χ2 = 27.45,P<0.0001** **Protective vs. Risk χ2 = 32.83,P<0.0001**
	Middle (29–34 yrs.)	92	0.72	0.18	0.1	
	Old (>34 yrs.)	62	0.67	0.21	0.12	

**N.B.** Chi-square test was performed between young vs. young, middle-age vs. middle age, and old age vs. old age groups from genotypic categories in a pair-wise fashion.

N = sample size

α = 0.05

**Table 7:** Frequency distribution of observed recombination among the MII error group stratified by maternal genotypes and maternal age group.

Genotype category of MII error group mothers	Age category	*N	Number of observed recombination			Chi-square value and P-value
			0	1	≥2	
MCM9 neutral genotype	Young (<29 yrs.)	26	NA	0.61	0.39	**Young (<29 yrs)-** **Neutral vs. Protective χ**^**2**^ **= 0.021,P = 0.8** **Neutral vs. Risk χ**^**2**^ **= 6.03,P = 0.01** **Protective vs. Risk χ2 = 4.71, P = 0.03**
	Middle (29–34 yrs.)	12	NA	0.7	0.3	
	Old (>34 yrs.)	20	NA	0.73	0.27	
MCM9 protective genotype	Young (<29 yrs.)	11	NA	0.63	0.37	**Middle (29–34 yrs)** **Neutral vs. Protective χ**^**2**^ **= 0.023, P = 0.8** **Neutral vs. Risk χ**^**2**^ **= 2.7,P = 0.1** **Protective vs. Risk χ**^**2**^ **= 3.22,P = 0.07**
	Middle (29–34 yrs.)	9	NA	0.69	0.31	
	Old (>34 yrs.)	9	NA	0.68	0.32	
MCM9 risk genotype	Young (<29 yrs.)	15	NA	0.78	0.22	**Old (>34 yrs)** **Neutral vs. Protective χ2 = 0.38, P = 0.5** **Neutral vs. Risk χ2 = 3.64,P = 0.04** **Protective vs. Risk χ2 = 7.12,P = 0.008**
	Middle (29–34 yrs.)	11	NA	0.81	0.19	
	Old (>34 yrs.)	12	NA	0.85	0.15	

**N.B.** Chi-square test was performed between young vs. young, middle-age vs. middle age, and old age vs. old age groups from genotypic categories in a pair-wise fashion.

N = sample size

α = 0.05

Linear regression analyses were conducted using maternal age and MCM9 genotype status as the predictor variables and amount of recombination events as the outcome for each genotype group. The result showed that the absence of recombination was negatively correlated with maternal age in all three of the genotype classes (P = 0.01 for all the three groups). This observation is concordant with the ‘DS risk model’ proposed in previous studies on DS sample populations from the US [2] and India [6] and again re-establishes the absence of recombination on 21q is maternal age-independent risk factor for Ch21 NDJ. In addition, logistic regression was performed considering maternal age, maternal MCM9 genotype, and the interaction between maternal age and maternal MCM9 genotype as predictors, and the amount of recombination as the outcome variable. This was a ‘case-only’ analysis, controls were not used as we do not have recombination information for control samples. It was observed that maternal age (i.e., the young age group served as the reference group: P = 0.03 for middle and P = 0.0 for old age categories) and maternal genotypes (neutral genotype as reference and P = 0.0 for risk variant), both, were significant predictors for the presence or absence of recombination, however the interaction term between these two, (i.e., age X genotypes) was not.

#### MII error group

A similar analysis was conducted for the MII error group women. Most of the risk variants did not exhibit a significant association with MII error except rs13647106147 T>G (Table 3). Despite this, stratification of the MII women followed the same definition of genotype categories used for MI women. This was done for two reasons. First, all recombination errors, (i.e., erroneous amounts of exchange or misplaced chiasma), arise during the pachytene stage of MI, and this is the only stage of cell division where chromosomes experience recombination abnormalities and segregate stochastically, resulting in aneuploidy. Thus, it could be considered that any genotype which poses a risk for aberrant recombination, and subsequent missegregation at MI is also an intuitive risk for MII errors. Second, the relatively small sample sizes of the MII category prevent us from detecting any significant statistical differences from the controls for the respective MCM9 risk genotypes. This is supported by the observation that some genotypes, which are considered a ‘risk’ for MI error (Table 2) were also more frequent among the MII women when compared with the controls, though they were statistically insignificant (Table 3). With the same logic, we consider polymorphisms as ‘protective’ towards recombination anomaly for MII women as it was observed in MI women.

The analyses of MII cases (Table 7) showed the same trend of reduced frequency in single and double observed recombination events across all age groups within the MCM9 ‘risk’, ‘neutral’ and ‘protective’ genotype categories. The estimate of ≥2 observed recombination events for the MCM9 ‘risk genotype’ group was 0.22 for the young, 0.19 for the middle and 0.15 for the old age groups respectively, whereas the estimate for the MCM9 ‘neutral genotype’ group was 0.39 for the young, 0.3 for the middle and 0.27 for the old age groups, and finally, for the MCM9 ‘protective genotype’ the frequency was 0.37 for the young, 0.31 for middle and 0.32 for the old age group. Similar to the observations of the MI error groups the frequency distributions of observed recombination between MCM9 ‘neutral’ and MCM9 ‘protective genotypes’ for all the age categories was not statistically significant (P = 0.8 for young age ‘neutral’vs.‘protective’), (P = 0.8 for middle age ‘neural’ vs.‘protective’) and (P = 0.5 for old age ‘neutral’ vs.‘protective’) (Table 7). However, the change in recombination frequency was significant for the young and old age groups having the risk genotypes with the middle age group being the exception in this instance (P = 0.1 for ‘neutral’vs.‘risk’, P = 0.07 for ‘protective’vs.‘risk’). Interestingly, an increased frequency of single recombination events (85%) and subsequent fewer amounts of two or more recombination events were observed in the older age group with ‘risk genotypes’when compared to females with ‘neutral’ (73%) and ‘protective genotypes’ (68%) (Table 7). This observation suggests that ‘risk genotypes’ probably reduce the frequency of two or more recombination events on the chromosomes that missgeregrate at MII. Logistic regression was conducted considering maternal age and genotypes as predictors and the amount of observed recombination as the outcome variables and the results were statistically insignificant. The interaction model (i.e., ‘maternal age X maternal genotype’) was also tested in the MII group and the effect remained insignificant.

### MCM9 variants and their relationship with the spatial distribution of single recombinant events on Ch21

#### MI error group

To study the effects of maternal MCM9 variations on the positioning of single exchange along the maternal Ch21q, we divided the entire length of 21q into six nearly equal intervals and identified the central placement of single recombinant events as a function of MCM9 genotype variants. This analysis was stratified by maternal age (as defined in the previous section) (Table 8). All three maternal genotype categories, namely MCM9 ‘neutral’, MCM9 ‘protective’ and MCM9 ‘risk genotypes’ exhibited concordant patterns of the placement of single observed recombination events similar to previous observations [2,6], (i.e., excess telomeric recombination events amongthe young age group with a gradual shift towards the middle of the chromosome arm with progressing age). For example, among q-arm a single recombinant events, 0.74, 0.77 and 0.74 occurred in telomeric intervals 5 and 6 (between markers D21S267 and D21S1446, spanning a region of ~8.2 Mb) among the young MI ‘neutral’, ‘protective’ and ‘risk’ genotype bearing women, respectively (Table 8). We observed a shifting of chiasma position towards the middle of the chromosome arm, into the intervals 3 and 4 (~15Mb between the markers D1S1257 and D21S167) among the older age group. This trend was significant in all three genotype categories as examined by linear regression models when maternal age was considered as a predictor variable (P = 0.00 for all the tested models), but not in the case of maternal genotype (P = 0.1). Furthermore, no statistically significant difference was observed among the distribution of recombination events among women with neutral, protective, and risk genotypes for any of the age categories (Table 8).

**Table 8:** Spatial distribution of observed single recombination events among the MI errors stratified by maternal genotype and maternal age.

			Frequency of single recombination events along Ch21q Centromere →Telomere						Average interval	Chi square & P value
Genotype category of MI error group mothers	Age category	N	Interval 1	Interval 2	Interval 3	Interval 4	Interval 5	Interval 6		
MCM9 Neutral genotypes	Young (<29 yrs.)	92	0.02	0.04	0.07	0.13	0.34	0.4	5.5	**Young (<29 yrs)-** **Neutral vs. Protective χ**^**2**^ **= 8.7,P = 0.11** **Neutral vs. Risk χ**^**2**^ **= 0.89,P = 0.9** **Protective vs. Risk χ**^**2**^ **= 0.53,P = 0.9**
	Middle (29–34 yrs.)	81	0.04	0.06	0.08	0.29	0.43	0.1	4.9	
	Old (>34 yrs.)	63	0.05	0.07	0.11	0.43	0.24	0.1	4.2	
MCM9 Protective genotypes	Young (<29 yrs.)	72	0.02	0.035	0.064	0.17	0.37	0.39	5.5	**Middle (29–34 yrs)-** **Neutral vs. Protective χ**^**2**^ **= 0.9,P = 0.9** **Neutral vs. Risk χ**^**2**^ **= 0.8,P = 0.9** **Protective vs. Risk χ**^**2**^ **= 2.38,P = 0.7**
	Middle (29–34 yrs.)	54	0.036	0.064	0.079	0.24	0.45	0.13	4.8	
	Old (>34 yrs.)	40	0.05	0.06	0.13	0.44	0.26	0.06	4.1	
MCM9 Risk genotypes	Young (<29 yrs.)	144	0.01	0.03	0.07	0.16	0.36	0.38	5.3	**Old (>34 yrs)-** Neutral vs. Protective **χ**^**2**^ **= 1.33,P = 0.9** Neutral vs. Risk **χ**^**2**^ **= 3.2,P = 0.66** Protective vs. Risk **χ**^**2**^ **= 1.3,P = 0.9**
	Middle (29–34 yrs.)	92	0.03	0.06	0.09	0.33	0.38	0.11	4.2	
	Old (>34 yrs.)	62	0.04	0.08	0.1	0.46	0.28	0.04	4.1	

N.B. Pairwise Chi-square test were performed to compare age groups:.young vs. young, middle vs. middle, and old vs. old for each genotypic category.

N = Sample size,

α = 0.05

#### MII error group

We observed (Table 9) the displacement of single recombination events from the middle of Ch21q in the younger women towards the centromere proximal position in older women across all the genotypic categories this pattern again confirms the previous observations [2,6]. The frequencies of single observed recombinant events among younger women from the ‘neutral’, ‘protective’ and ‘risk’ genotype groups were 0.66, 0.64, and 0.68 respectively, in the intervals 3 and 4 (~15Mb between the markers D1S1257 and D21S167). In contrast, the older women from all the genotype categories i.e., ‘neutral’, ‘protective’, and ‘risk’ displayed 0.66, 0.65, and 0.7 single recombination events respectively, in the intervals 1 and 2 (~3.5Mb segment between D21S369 and D21S214). Using linear regression models we confirmed maternal age as a significant predictor of recombination position (P = 0.00 for all the models tested), but not the maternal genotypes. The absence of a relationship between the maternal MCM9 genotypes and the distribution of single recombination events on 21q was also confirmed by pairwise Chi-square test between the age groups from the various genotype categories (Table 9).

**Table 9:** Spatial distribution of observed single recombination events among women displaying MII errors stratified by maternal genotype and maternal age.

			Frequency of single recombination events along Ch21q Centromere →Telomere						Average interval	Chi square & P value
Genotype category of MII error group mothers	Age category	N	Interval 1	Interval 2	Interval 3	Interval 4	Interval 5	Interval 6		
MCM9 Neutral genotype	Young (<29 yrs.)	26	0.07	0.1	0.33	0.33	0.16	0.01	3.5	**Young (<29 yrs)-** **Neutral vs. Protective χ2 = 0.43, P = 0.66** **Neutral vs. Risk χ2 = 0.24, P = 0.81** **Protective vs. Risk χ2 = 0.19, P = 0.85**
	Middle (29–34 yrs.)	12	0.12	0.33	0.27	0.15	0.1	0.03	3.2	
	Old (>34 yrs.)	20	0.37	0.29	0.18	0.1	0.05	0.01	2.5	
MCM9 Protective genotype	Young (<29 yrs.)	11	0.05	0.11	0.29	0.35	0.17	0.03	3.3	**Middle (29–34 yrs)-** **Neutral vs. Protective χ2 = 0.17,P = 0.864** **Neutral vs. Risk χ2 = 0.36, P = 0.72** **Protective vs. Risk χ2 = 0.71, P = 0.86**
	Middle (29–34 yrs.)	9	0.11	0.36	0.29	0.14	0.09	0.01	3.1	
	Old (>34 yrs.)	9	0.35	0.30	0.19	0.11	0.04	0.01	2.4	
MCM9 Risk genotype	Young (<29 yrs.)	15	0.03	0.11	0.35	0.33	0.16	0.02	3.4	**Old (>34 yrs)-** **Neutral vs. Protective χ2 = 0.19, P = 0.84** **Neutral vs. Risk χ2 = 0.84, P = 0.4** **Protective vs. Risk χ2 = 0.51, P = 0.61**
	Middle (29–34 yrs.)	11	0.1	0.35	0.24	0.17	0.09	0.05	3.0	
	Old (>34 yrs.)	12	0.4	0.3	0.15	0.1	0.03	0.02	2.1	

N.B. Pair wise chi square test were performed to compare age groups: young vs. young, middle vs. middle and old vs. old from all the genotypic categories.

N = Sample size,

α = 0.05

### *In silico* analyses of polymorphic alleles

*In silico* analyses are generally done to anticipate the probable damaging effects of polymorphisms or mutations of interest in advance of wet lab experiments. We conducted a ‘pilot study’ to predict the imperilments incurred by ‘risk variants’ at the transcript or protein level of MCM9 expression, and their association with Ch21 nondisjunction. For this study, we analyzed all 25 risk variants using the *Mutationt@ster* and *Human Splice Finder programs*. The exonic variants that caused missense amino acid replacements were tested using the *PROVEAN*, *SIFT*, *and PolyPhen-2* programs which are designed to interpret the effects of SNPs on protein sequence, structure, and function. We used multiple programs for this analysis as the simulating software have been designed using different interfaces and algorithms to infer the damaging effects of a given mutation or polymorphism. However, the outcomes of these programs are sometimes contradictory. We considered a given variant as ‘fatal’ when at least two programs detected it as damaging. The summary of the outcomes of the analyses through all five programs is presented in Table 10.

**Table 10:** Summary of *in silico* analyses of the twenty-five polymorphic risk variants for MI errors.

Variant	Type	Amino acid change	Mutation taster	Human splice finder	Polyphen2	SIFT	PROVEAN
rs114000233 T>G	Promoter variant	Non-coding	Disease causing	Alteration of enhancer site; Deletion of silencer site	-	-	-
rs62422268 C>G		Non-coding	Polymorphism	Alteration of enhancer site	-	-	-
rs62422266 T>C		Non-coding	Polymorphism	Creation of new silencer site	-	-	--
MK647975 G>A	Novel, Exonic (Exon 2), Synonymous	Lysine	Disease causing	Deletion of splice acceptor, Deletion of exonic enhancer site, Creation of new exonic silencer site	-	-	-
MK647977 G>A	Novel, Exonic (Exon 2), Synonymous	Glutamic acid	Disease causing	Creation of new exonic silencer site	-	-	-
rs73521381 T>G	Exon 2, Synonymous	Serine	Polymorphism	Creation of new exonic silencer site	-	-	-
rs1267215855 T>C	Exon 3, Synonymous	Proline	Disease causing	Deletion of existing splice acceptor, donor site and branch point, Creation of new splice acceptor site, donor site and branch point, new enhancer and silencer site created	-	-	-
rs1331061317 G>A	Exon 3, Synonymous	Glutamic acid	Disease causing	Alteration of splice acceptor site, deletion of existing splice branch point, deletion of existing enhancer site and creates alternate enhancer site	-	-	-
rs375494814 T>C	Exon 3, Synonymous	Serine	Disease causing	Deletions of existing splice acceptor and enhancer sites, altering of splice donor site. Creation of new enhancer and silencer sites	-	-	-
rs1486475303 C>A	Exon 3, Synonymous	Tyrosine	Disease causing	Deletion of existing donor site, enhancer site and silencer site. Creation of a new silencer site	-	-	-
rs573678849 G>A	Exon 3, Synonymous	Glutamic acid	Disease causing	Deletion of existing silencer site. Creation of new silencer site	-	-	-
rs370725680 T>C	Exon 3, Synonymous	Serine	Disease causing	Deletion of existing splice acceptor and enhancer site. Creation of new silencer site	-	-	-
MK647976 G>A	Novel, Exonic (Exon 2), Missense	Aspartic acid-Asparagine	Disease causing	Creation of new silencer site	Benign	Damaging	Neutral
MK647978 G>C	Novel, Exonic (Exon 2), Missense	Alanine-Proline	Disease causing	Deletion of existing splice donor site, Creation of new exonic enhancer site	Damaging	Damaging	Neutral
rs531682044 G>A	Exon 2, Missense	Arginine-Glutamine	polymorphism	Creation of new exonic enhancer site	Benign	Tolerated	Neutral
rs140838152 A>G	Exon 3, Missense	Glutamic acid-Valine	Disease causing	Deletion of existing donor site, alteration of branch point. Creation of new silencer site	Damaging	Damaging	Deleterious
rs576382724 A>C	Exon 3, Missense	Histidine-Proline	Disease causing	Creation of new acceptor, donor and silencer site. Alteration enhancer site	Benign	Tolerated	Deleterious
rs367896634 G>C	Exon 3, Missense	Arginine-Glutamine	Disease causing	Creation of new enhancer site	Damaging	Damaging	Deleterious
rs1322432805 G>A	Exon 3, Missense	Lysine	Disease causing	Does not affect splice site	Damaging	Damaging	Deleterious
rs1316687536 T>G	Exon 3, Missense	Valine-Alanine	Disease causing	Creation of new splice acceptor site	Benign	Damaging	Deleterious
rs1364710617 T>G	Exon 3, Missense	Cysteine-Arginine	Disease causing	Deletion of existing enhancer site	Damaging	Damaging	Deleterious
rs754872940 A>C	Exon 3, Missense	Histidine-Arginine	Disease causing	Creation of new enhancer site while deletion of existing silencer site	Benign	Tolerated	Deleterious
rs549531759 G>A	Exon 3, Missense	Glutamine-Histidine	Disease causing	Creation of new splice acceptor site. Altering of existing enhancer site to silencer site	Damaging	Damaging	Deleterious
rs764822140 C>A	Exon 3, Stop gained	Non coding	Disease causing	Deletion of existing donor site. Creation new enhancer site	NA	NA	NA
rs1370486625 G>T	Intron variant	Non coding	Polymorphism	Deletion of existing splice acceptor site and creates new acceptor site; Creates new Intronic silencer site	NA	NA	NA

Note- PROVEAN, SIFT and PolyPhen 2 can only predict outcome for missense variants.

### Predictions from ‘PolyPhen-2’ (Polymorphism Phenotyping v2)

We screened the eleven exonic missense variants namely MK647976 G>A, MK647978 G>C, rs531682044 A>G, rs140838152 A>G, rs576382724 A>C, rs367896634 G>C, rs1322432805 G>A, rs1316687536 T>G, rs1364710617 T>G, rs754872940 A>C and rs549531759 G>A (Refer to S1 Table). No predicted outcomes were generated for the synonymous variants. Five, out of the eleven missense variations [i.e., nsSNPs] MK647976 G>A, rs531682044 G>A, rs576382724 A>C, rs1316687536 T>G and rs754872940 A>C were predicted as ‘**BENIGN**’ with their respective HumVar scores 0.006, 0.007, 0.249, 0.228, and 0.014, respectively. The remaining six variants, MK647978 G>C, rs140838152 A>G, rs367896634 G>C, rs1322432805 G>A, rs1364710617 T>G and rs549531759 G>A, were predicted as ‘**PROBABLY DAMAGING**’ with HumVar scores 0.884, 0.998, 1, 0.997, 1, and 0.994.

### Predictions from ‘Mutationt@ster’

The program detected the non-coding variants rs62422268, rs62422266, and rs1370486625 located within the promoter and intron as ‘**POLYMORPHISMS**’. However, one promoter variant rs114000233, was predicted as ‘**DISEASE CAUSING**’. Interestingly, the novel alleles MK647975, MK647976, MK647977, and MK647978 were all predicted as ‘**DISEASE CAUSING**’. In addition, many variants found within exon 3, namely rs126721585, rs1331061317, rs375494814, rs1486475303, rs573678849, rs370725680, rs140838152, rs576382724, rs367896634, rs1322432805, rs1316687536, rs1364710617, rs754872940, rs549531759 and rs764822140 were all predicted as ‘**DISEASE CAUSING**’. However, two of the coding region variations, rs531682044 and rs73521381 were predicted as ‘**POLYMORPHISMS**’ (S1 Table).

### Predictions from ‘Protein Variation Effect analyzer (PROVEAN)’

We screened all the eleven exonic missense variants, namely MK647976 G>A, MK647978 G>C, rs531682044 A>G, rs140838152 A>G, rs576382724 A>C, rs367896634 G>C, rs1322432805 G>A, rs1316687536 T>G, rs1364710617 T>G, rs754872940 A>C and rs549531759 G>A, with this program (S1 Table). No impact of the synonymous variants on MCM9 protein structure and function was predicted. Three variants MK647976, MK647978 and rs531682044 were predicted as ‘**NEUTRAL**’ with scores -0.648, -2.456 and -1.124 respectively. The remaining eight variants, rs140838152 A>G, rs576382724 A>C, rs367896634 G>C, rs1322432805 G>A, rs1316687536 T>G, rs1364710617 T>G, rs754872940 A>C, and rs549531759 G>A were all predicted as ‘**DELETERIOUS**’ with scores -5.96, -4.51, -3.69, -4.51, -3.06, -11.31, -4.81, and -3.77, respectively.

### Prediction from ‘Sorting Tolerance From Intolerance’ (SIFT)

We screened all eleven exonic missense variants MK647976 G>A, MK647978 G>C, rs531682044 A>G, rs140838152 A>G, rs576382724 A>C, rs367896634 G>C, rs1322432805 G>A, rs1316687536 T>G, rs1364710617 T>G, rs754872940 A>C and rs549531759 G>A (Refer to S1 Table). SIFT identified five variants as ‘**TOLERATED**’: MK647976 G>A, MK647978 G>C, rs531682044 G>A, rs576382724 A>C, and rs754872940 A>C. The remaining six variants rs140838152 A>G, rs367896634 G>C, rs1322432805 G>A, rs1316687536 T>G, rs1364710617 T>G, and rs549531759 G>A were identified as “**DAMAGING**”.

### Prediction from ‘Human Splicing Finder’ (HSF)

We identified that (S2 Table) that the presence of minor allele ‘G’ of the SNP rs11400023 abolishes the intronic splicing silencer sequence and creates a new enhancer sequence (S2 Table). The SNP rs62422268 was found to create a new silencer sequence. The SNP rs62422266 was found to alter a splice enhancer site. The novel allele mutant “A” of the exonic variant MK647975 deletes the WT splice acceptor site. It deletes not only an acceptor site but also predicted to remove the auxiliary splice enhancer site while creating a new splicing silencer site. The novel variant MK647976 may create a new silencer site. HSF predicted that the mutant “A” allele for the novel site MK647977 deletes a splice branch point motif. Interestingly, presence of the ‘A’ allele also deletes the existing splice enhancer sequences. Further, the HSF predicted that the novel minor allele “C” of MK647978 deletes the splice donor site. The minor allele “C” is predicted to create a new enhancer site as well as a silencer site. The HSF predicted that the mutant ‘T’ allele of rs1370486625, deletes the existing splice acceptor sequence and creates a new splice acceptor site instead. The software also predicted a new splice silencer motif that overlaps with the new acceptor site. The software generated a splicing enhancer signal for the rs531682044. The minor allele “G” of the variant rs73521381 generates two silencer motifs. The minor allele “C” of rs1267215855 breaks the existing splice acceptor site while creating a new site.

The minor allele “A” of rs1331061317 alters the existing splice acceptor site, deletes the existing branch point while creating a new alternate branch site. The minor allele “C” deletes the existing splice acceptor site and alters the existing donor site. It also deletes the existing splice enhancer site and creates a new enhancer site and a new silencer site. The minor allele “A” of rs1486475303 breaks the existing splice donor, enhancer, and silencer site while creating a new splice silencer site. The minor allele “A” for rs573678849 deletes the existing silencer site and creates a new silencer site. The minor allele “C” of the variant rs370725680 deletes the existing splice acceptor and enhancer site while creating a new silencer site. The minor allele “G” of variant rs140838152 deletes the existing splice donor site, alters the branch point, and creates a new silencer site. The minor allele “C”of the variant rs576382724 creates a new splice acceptor and donor site, deletes the existing enhancer, and creates a new enhancer and silencer sites. The minor allele “C” of rs367896634 creates a new silencer site. The HSF predicted no effect of the polymorphism rs1322432805 on the splicing process. The minor allele “G” of rs1316687536 creates a new splice acceptor site. The minor allele “G”of rs1364710617 deletes the existing splice enhancer site. The minor allele “C” of variant rs754872940 creates a new enhancer site while breaking the existing silencer site. The minor allele “A” of rs549531759 creates a new acceptor site, deletes the existing enhancer site and creates a new silencer motif. Finally, HSF predicted the deletion of a splice donor site and creation of a new silencer motif in the MCM9 transcript due to minor allele “A” of the variant rs764822140.

## Materials and methods

### Ethics statement

The study was conducted following the principles outlined in the Declaration of Helsinki and was approved by the institutional ethics committee constituted by the University of Calcutta and Institute for Post Graduate Medical Education and Research (IPGMER) Kolkata. Written consent in the pre-printed questionnaire was taken from each family for participation in the study.

### Selection of study subjects

All the participating families were recruited into the study following their initial reporting to the Pediatrics Department of IPGMER, Kolkata, and subsequent diagnosis of the child as a suspected DS cases. Initially, 1007 families were selected for inclusion in the study due to their complete family history and epidemiological detail. A total of 825 mothers who gave birth to karyotype-confirmed children with Down syndrome were included in the study. Simultaneously, 855 age-matched mothers with a euploid child were chosen from the same hospital registry as the ‘control group’ to maintain maximum demographic and socio-economic similarities with cases. Women in the control population had no history of miscarriage or abnormal pregnancy. All participating mothers (both cases and controls) belonged to the same geographical location and ethnic group which ensured genetic homogeneity among the population. All participating mothers were interviewed and their epidemiological details and all relevant data were recorded in pre-printed form.

### Collection of samples

Venous blood samples (2ml) were collected from the participating mothers only after obtaining their full consent. Samples were collected in EDTA coated vacutainer tubes and stored at -20°C until genotyping was completed. The highest biomedical ethics were enforced during this work.

### Karyotyping

The free trisomy 21 status of all the children included in the molecular study was confirmed by classical karyotyping. At least 40 G-banded metaphase plates for the trios were analyzed for each case family.

### Determination of parental and meiotic origin of supernumerary Ch21

A set of thirty-two short tandem repeat (STR) markers specific to 21q (centromere-D21S369-D21S215-D21S258-D21S120-D21S1431-D21S1904-D21S192-D21S1432-D21S11-D21S1437-D21S2053-D21S1884-D21S214-D21S1257-D21S1914-D21S265-D21S210-D21S1270-D21S226-D21S1908-D21S224-D21S167-D21S1222-D21S267-D21S1412-D21S2055-D21S168-D21S212-D21S1260-D21S1890-D21S1903-D21S1446-telomere) were used to determine of the parental origin of the supernumerary Ch21 via PCR. A subset of five peri-centromeric makers (D21S369-D21S215-D21S258-D21S120-D21S1431) was used to interpret the meiotic stage of error i.e., either meiosis [MI] or meiosis [MII]. When the maternal heterozygous alleles of a given marker were inherited by the child (i.e., the heterozygousity or two allelic state in mother was maintained in the next generation) we inferred the stage of error as MI. When the heterozygous maternal alleles were found inherited in the reduced state in the child with DS (i.e., two alleles in the mother were reduced to a single allele in the child with DS) we infered the stage of error as MII.

### Estimation of recombination along 21q

Using the above-mentioned set of STR markers, family data was arranged in a data string as NNNNRRRUUR (i.e., ‘N’ for non-reduced state of the markers, ‘R’ for reduced state of the markers and, ‘U’ for unidentified state of the markers). Briefly, a single recombination event was counted when a change in the status of two successive markers from non-reduction (N) to reduction (R) or vice versa was observed. These methods have been described elsewhere [24]. When recombination was scored at the junction of two adjacent divisions then it was considered as equally likely to occur in both segments. For uninformative markers (U), the detected recombinant event was distributed evenly betweenthe respective intervals.

### Genotyping

#### Gene region selection and primer designing

Specific primers (S3 Table) were designed and used to amplify the MCM9 gene [transcript MCM9-202, ID- ENST00000316316.10, CCDS56447]. For this purpose, we used the NCBI Conserved Domain Search (*ncbi*.*nlm*.*nih*.*gov*). Multiple ATP binding sites span from the 314V residue to the 567R residue and this region corresponds to exons 6, 7, 8, 9, and 10. Out of the total 13 exons, we selected the promoter region (ENSE00002068426), exon 2(ENSE00002046152), 3 (ENSE00001160217), 5 (ENSE0000840062), 6 (ENSE00000840059), 7 (ENSE00001722724), 8 (ENSE00003683120), 9 (ENSE00003504738), 10 (ENSE00001160221) and 13 (ENSE00001447244) for variant screening, while exons 4 (ENSE00000840063), 11 (ENSE00001512182) and 12 (ENSE00001512181) were not taken into account due to the lack of variants. While selecting exons and promoters, some flanking intronic sequences and upstream promoter regions were also included to effectively cover the entire exon. The primers were designed by using Primer3 software (*primer3*.*ut*.*ee*) and were supplied by the manufacturer Integrated DNA Technologies (IDT).

#### Polymorphism analysis

Genomic DNA was isolated from the whole blood samples using the QIAGEN QIAamp Blood Mini Kit (Catalogue No. 51104) per the manufacturer’s instruction and stored at -20°C until further analysis. To detect single nucleotide polymorphism [SNPs], genotyping was carried out by Polymerase Chain reaction (PCR) followed by direct Sanger Dye Deoxy sequencing. PCR amplification was conductedin a 30 μl volume containing 50–100 ng of DNA, 1 μl of each primer (10 mmol/L), 0.2 μl of deoxyribonucleotide triphosphate mix (dNTPs, 10mmol/L; Invitrogen Carlsbad, CA, USA), 1.5 μl magnesium chloride (MgCl2, 50 mmol/L), 1X PCR reaction buffer and 0.8 μl of Taq Polymerase (5 units/lμl; Invitrogen, California, USA). PCR products were directly sequenced using a Taq Dye Deoxy Terminator sequencing kit (Applied Biosystems, Foster City, USA) with an ABI Prism 377 DNA Sequencer (Applied Biosystems, Foster City, USA). The electropherogram and DNA base sequence was viewed using the program Snap-Gene Viewer (version 5.0.7).

### *In silico* analyses

We used five programs to predict the damaging effects of the polymorphic variants on MCM9 mRNA transcripts and proteins. The softwares are PolyPhen 2, Mutationt@ster, PROVEAN, SIFT (Sorting Tolerance From Intolerance) and Human Splicing Finder (HSF).

PolyPhen 2 specifically targets nonsynonymous/missense (nsSNPs), (i.e., SNPs located within the coding regions (CDS) that alters amino acids in translated gene products [25–28]).PolyPhen 2 generates two pairs of data sets. The first pair, “HumDiv” is compiled from all damaging alleles with known effects on the molecular function causing human Mendelian diseases, present in the UniPortKB database. The second pair, “HumVar”, consists of all human disease-causing mutations from UniPortKB, together with common human nsSNPs [Minor Allele Frequency (MAF) >1%] without an annotated involvement in disease. A mutation is appraised qualitatively, as ‘**BENIGN**’, ‘**POSSIBLY DAMAGING**’, or ‘**PROBABLY DAMAGING**’ based on pairs of false-positive rate (FPR) thresholds, optimized separately for each model (e.g., threshold value = 5/10% for HumDiv and 10/20% for HumVar). Mutations with their posterior probability scores associated with estimated false-positive rates at or below the first (lower) FPR value are predicted to be ‘**PROBABLY DAMAGING**’ (more confident prediction). Mutations with the posterior probabilities associated with false-positive rates at or below the second (higher) FPR value are predicted to be ‘**POSSIBLY DAMAGING’** (less confident prediction). Mutations with estimated false-positive rates above the second (higher) FPR value are classified as ‘**BENIGN’**.

The *Mutationt@ster* predicts the disease-causing potential of a nucleotide alteration by using Bayes classification [29]. The software automatically generates three different models aimed at different types of alterations, ‘silent’ (non-synonymous or intronic) (without_aae model), substitution/insertion/deletion of a single amino acid (simple_aae model), or more complex changes of the amino acid sequence (e.g. mutations introducing a premature stop codon, etc—complex_aae model). The output value is the probability of the prediction. A value close to ‘1’ indicates a high ’security’ of the prediction. Alterations causing a premature termination codon and ultimately leading to nonsense-mediated mRNA decay (NMD) are assigned the ‘**DISEASE CAUSING**’ status while otherwise assigned as ‘**POLYMORPHISM**’.

This online software predicts whether a single/multiple amino acid substitution/substitutionsor ‘indel’ has any implications on the normal biological functioning of the protein generated from the reading frame [30–32]. This software initially clusters specific “BLAST hits” [The “BLAST hits” is performed by “CD-HIT program (cluster peptide sequences)”[33]] and generates a ‘**supporting sequence set**’ that has similarity in sequence. A ‘**delta alignment score**’ is calculated for each ‘**supporting sequence**’ and the average of the alignment scores within and across the clusters generates the final PROVEAN score. If the PROVEAN score is equal to or below the threshold (i.e., -2.5), the variant is predicted to have a ‘**DELETERIOUS**’ effect and if the score is above the threshold, the variant is predicted to have ‘**NEUTRAL**’ effect. PROVEAN is useful only for evaluating nonsynonymous variants (nsSNPs) or indel variations [i.e., insertion or deletion].

The SIFT program predicts the effect of amino acid substitutions on protein function. This prediction uses sequence homology and the physical properties of amino acids. SIFT can be applied to naturally occurring nonsynonymous polymorphisms and laboratory-induced missense mutations.

To understand the effect of intronic and well as exonic mutations leading to splicing defects we screened our variants using the Human Splicing Finder software [34] (Refer to S2 Table). The Human Splicing Finder (HSF) uses different algorithms to identify and predict mutations’ effects on splicing motifs including acceptor and donor splice sites, the branch point, and auxiliary sequences known to either enhance or repress splicing: Exonic Splicing Enhancers (ESE) and Exonic Splicing Silencers (ESS). These algorithms are based on ‘Position Weight Matrices (PWM)’, ‘Maximum Entropy principle’, and the ‘Motif Comparison method’. There is a predefined threshold consensus value and score variation value for each algorithm. The software compares the wild type (WT) and mutant score values with the set threshold cut-off. When a mutation occurs, if the WT value is above the threshold, while the mutant value is below, it is considered a splice site disruption, whereas, if the mutant value is above the threshold while the WT value is lower, it is predicted as a new site.

### Statistical analyses

All the statistical analyses were were conducted using SPSS version 25 and significance was tested at a 95% confidence level. We employed Fisher’s Exact Test to compare MI: MII ratios, t-tests were used to test for differences between mean parental ages. Chi-square tests were used to compare various age-stratified recombination frequencies among the MCM9 genotype classes in pairwise tests. Linear regression was used to evaluate the trend of the relationship between maternal age and both the amount and location of recombination as well as the position of single observed recombinant event. In the regression analysis, the maternal age group was used as the independent variable and the amount of recombination served as the outcome variable. Additionally, logistic regression was performed keeping maternal age, maternal genotype, and maternal age X maternal genotype as predictor variables and the amount of recombination as the outcome variable.

## Discussion

Altered recombination has been identified as a risk factor for Ch21 NDJ over the years [2,6,35], but the underlying cause remain elusive. It may be possible that both environmental insults and genetic risk factors challenge the occurrence of ideal recombination patterns between meiotic homologues. Previously we [6,36] and others [2] identified both the reduction in the amount of observed recombination and the altered placement of recombination on Ch21 as risk factors for have a child with DS. Now we have taken a candidate gene approach to characterize the etiological factors behind the meiotic exchange error in oocytes. The MCM9, in association with other proteins regulates homologous recombination and maintains genomic integrity [14,15,17–19]. We have analyzed polymorphic variants within this gene among women having a child with DS, as well as among controls from Bengali-speaking populations from the eastern part of India. We were curious to determine whether the polymorphic alleles of MCM9 affect the amount and location of meiotic recombination on Ch21, and whether this is damaged by maternal age. Out of the forty-one SNPs that we identified in the reading frame of MCM9 gene (Table 1), twenty-five sites with thirty-eight maternal genotypes exhibited a significant association with maternal MI errors. On the contrary, for eight SNPs we determined that the minor allele was more prevalent among the mothers with healthy euploid children (i.e., the control group). We therefore infer that these twenty-five variants serve as genetic risk factors for MI NDJ in oocytes in the Bengali population and that the eight SNPs are potentially protective against NDJ errors. To inquire whether these variants are inherited together as haplotypes, we performed LD analyses. We inferred that these susceptible genotypes exhibit a weak linkage as we found R^2^ = 0.07. As the physical distance between these sites on the MCM9 reading frame is only ~21Kb, which is much less than the needed minimum distance of >50–60 Kb between two loci to achieve correct result from a given program [37], we did not obtain any significant value in LD analysis. We can only infer that the polymorphic sites exhibit a strong association with each other and impart risk a for MI errors and the occurrence of these three genotypes among the women was probably stochastic. Additionally, we performed model analyses to test the dominant and additive effects of alleles and combined genotypes as the risk of MI NDJ errors (Table 3). Some of the models exhibited significant additive effects, however verified among other populations.

We observed an association of all twenty-five risk variants only with the MI error, and not with the MII error. Considering the biology of MCM9 helicase activity during meiosis I, it is not difficult to understand its association with MI errors. Based on the findings of our study, we hypothesize that the defects in MCM9 wild type proteins or imbalances in the ratio of its different isoforms may lead to a reduction in exchange at MI, and NDJ occurs. When stratified with the maternal age categories, we observed a reduction in detectable exchange across all the age groups who bear MCM9 risk variants. Though the reduction in recombination is a generalized feature for nondisjoined Ch21, we scored ~21%, ~22%, and ~33% increase in non-recombinant meiosis among the MCM9 risk genotype women for MI young, middle and old age groups, respectively (P<0.0001 for young, P = 0.0023 for middle and P = 0.0024 for older, Table 5). This is a very unique observation as it suggests the non-stochastic occurrence of lack of exchange between meiotic homologues and specific quantum of the contribution of MCM9 variants in recombination error on one hand and the presence of other risk factors (both stochastic and other genetic/molecular challenges) associated with achiasmate meiosis among the MCM9 protective and neutral variant DS bearing women on other hand. Even, we observed fewer amounts of detectable double recombinant events in the MII category, too (Table 6). We scored ~13%, ~10%, and ~6% less ≥2 events among the MII MCM9 risk genotype women of all age groups, though statistically significant result was obtained only for the old age group when comparison was done between MCM9 protective and neutral genotype categories. This needs careful rationalization. Though we observed a strong association of all twenty-five risk alleles with MI error, many women of the MII error group bear MCM9 risk variants too. For them, suboptimal functioning of MCM9 and subsequent drop in exchange frequency between homologues might have been compromised through MI in some way but not at MII, when the molecular backup system of chromosome seggeration apparatus in the overy of old age mother could not work properly and NDJ occurred. Alternatively, it may possible that the MCM9 risk alleles that obliterate crossing over may cause precocious separation of sister chromatids (PSSC) or reverse segregation, the processes which may be frequent than classical NDJ as evident from genome-wide recombination analysis of human oocyte in the study [38], which suggests typical non-exchange chromosome usually undergo PSSC or reverse segregation (with or without MII missegregation), instead of classical MI error. Again, this is an intuitive interpretation and needs an additional molecular study for confirmation. Reduction in cross over activity was evident in the study that has estimated the recombination of the genome of aneuploid human oocytes and polar bodies with a more sophisticated single-cell genome analysis technique [39].

We observed single telomeric recombinant events among the young mothers of the MI category and subsequent shift of single recombinant events towards the middle of 21q. This trend was found among the MCM9 risk, protective and neutral genotype women (Table 7). The frequency distribution of single recombinant events across the six intervals on 21q was found similar in all the genotype categories. On the other hand, we recorded a single pericentromeric recombinant events among the older women of all the three MCM9 genotype classes from the MII error group (Table 8) and the distribution remained statistically similar when compared in pairwise fashion. Both sets of MI and MII data suggest collectively that MCM9 mutations/polymorphisms may not be a risk for aberrant positioning of recombinant events on 21q.

Maternal age-stratified analyses for the number of exchange events among the three genotype classes under the MI and MII error groups revealed maximum non-exchange meiosis in the young group (<29 years) with a gradual decrease in the frequency with age and this trend is concordant to our previous observation [6]. Additionally, the mean maternal age of DS conception among MCM9 risk, protective and neutral genotype women was not different (Table 1). These two observations suggest that the NDJ risk in oocyte imposed by MCM9 polymorphisms/mutations is maternal age-independent and equally affect all the age groups.

Lastly, to infer the probable damaging effect of the MCM9 polymorphisms at the molecular level we employed *in silico* approach as genomic polymorphisms are known to influence the expression of the gene and may confer susceptibility to certain diseases or disorders. We ran five different programs. Among them ‘PolyPhen2’, ‘Mutationt@ster’, ‘PROVEAN’, and ‘SIFT’ were employed to predict the probable damages at MCM9 protein structure and function, whereas ‘Human Splice Finder (HSF)’ was used to predict the change in MCM9 transcript sequence that could affect splicing. The outcome of the programs may differ owing to different algorithms and interfaces used to design the programs. For the programs that analyzed change for defects in protein structure, we tested only the exonic variants. The *PolyPhen2* predicted MK647978, rs140838152, rs367896634, rs1322432805, rs1364710617 and rs549531759 as ‘**DAMAGING**’ whereas MK647976, rs531682044, rs576382724, rs1316687536, rs754872940 as ‘**BENIGN**’. *Mutationt@ster* predicted that twenty out of the twenty-five risk variants as ‘**DISEASE CAUSING**’. The *PROVEAN* detected eight missense variants in exon 3, namely rs140838152, rs5763896634, rs1322432805, rs1316687536, rs1364710617, rs754872940 and rs549531759 as ‘**DELETERIOUS**’. *SIFT* detected MK647976, MK647978, rs140838152, rs367896634, rs1322432805, rs1316687536, 1364710617 and rs549531759 as ‘**DAMAGING**’.

The outcome of ‘Human Splice Finder (HSF)’ is extensive. Mutations/variations within the splicing regulatory sequences are often implicated in various genetic disorders [40] and other diseases [41–43]. Exons [44] and introns [45] have highly conserved Splicing Regulatory Elements (SRE) that are *cis*-acting and either splicing enhancers or silencers [i.e., Exonic splicing enhancers (ESE), Exonic splicing silencer (ESS), Intronic splicing enhancers (ISE) and Intronic splicing silencers (ISS)] that refine bona fide exon recognition. Therefore, genetic variations in these cis-acting SREs result in anomalous intron excision and ultimately lead to varying protein products [46,47]. We tested all the twenty-five risk variants of MCM9 by this program. The result shows (Table S2) some variants delete pre-existing splice acceptor (example MK647975G>A), splice donor (example MK647978 G>C), enhancer, and silencer (example rs114000233 T>G) sites while some other variants creates new site (example rs1370486625). Some however alters the pre-existing splice sites. Only one variant rs1322432805 does not have any effect on splicing. All these results together suggest imperilments induced by change in proportions of different alternate splice variants of MCM9 transcript in the oocytes probably disrupt usual exchange pattern between Ch21 homologues in oogenesis and cause NDJ at MI. Again these predictions need confirmation through proper elaborate molecular analyses.

### Conclusion and future direction

In summary, we report, for the first time, that polymorphic variants of the recombination and DNA repair regulator helicase MCM9 gene are associated with an increased risks for recombination failure in meiosis and most MI errors involving Ch21. In addition, this phenomenon occurs in a maternal age independent manner. We did not find that the variations in MCM9 were associated with the altered placement of recombination on 21q. Further, we report some novel alleles on certain exonic polymorphic sites that are unique to the Indian Bengali population. Some of these variants are risk imposing whereas some are protective to NDJ. Collectively these findings suggest for the first time, the contribution of the MCM9 gene in proper chromosome segregation in the oocytes. Importantly, all twenty-five risk imposing polymorphic sites have been proven *in silico* as damaging toward optimal splicing of the MCM9 transcripts and generate aberrant splice variants that lead to faulty functioning of the protein.

A previous study [48] on Ch21 NDJ suggests the presence of certain trans-acting factors in the maternal genome that not only affect the recombination pattern on Ch21 but cause a global reduction in recombination on other chromosomes as well. It may possible that the MCM9 is one of the probable candidates of that hypothetical trans-acting factor, though estimation of recombination on other chromosomes in the oocyte from the MCM9 risk variant mothers was beyond the scope of the present study. Very recently, a GWAS [36] has been conducted on DS samples from the US population that reported a strong association of some of the maternal genomic loci either with MI or with MII errors within the oocytes. Intuitively, any or all these candidate genes may act as so-called “trans-acting” global regulators of genomic recombination profile. The outcome of our present study, together with the results obtained in the study by Chernus et al. [36], strengthen the notion that genetic risk that predisposes mother to have Ch21 errors in the oocytes is multifactorial and various trans-acting factors determine the optimal and delicate balance of proteins necessary for faithful chromosome segregation.

Our study suffers from some limitations; first we have not estimated potential exchanges between Ch21 homologues at the four-strand stage and thus the fraction that we designated as ‘observed non-recombinant group’ under the MI category is possibly overestimated and does not representthe non-exchange group (E0) as scored in other studies [38]. Secondly, we were not in the position to distinguish PSSC or reverse segregation from classical MI NDJ errors with the STR-genotyping method and possibly grouped them within the MI error category. This would be most probable as the previous study [38] suggested E0 oocytes often experience PSSC or reverse segregation than classical MI NDJ. Third, we could not explore the effect of the MCM9 risk variants on the recombination profile of Ch21 from siblings of the trisomy 21 proband. Most of our mothers had their first child with DS and reported randomly to our laboratory immediately following birth and did not return. So their later reproductive outcome remained unknown to us. Only a few parents with one or more elder siblings of the proband participated in our study and did not consent for donating tissue of their healthy euploid child due to social reasons. Besides, comparatively smaller sample size limited our attempt to resolve the relationship between the MCM9 variants and the MII error group andour inability to perform NGS restricted from finding deep sited polymorphisms and copy number variations in MCM9 in the maternal genome. Also, the *in silico* predictions are theoretical, and even though they give a clear idea about the harmful influence of the variations, a proper *in vivo* approach is essential to validate these predictions. Nevertheless, our work provides the foundation for future study to characterize similar candidate genes that work conjointly with MCM9 to regulate recombination in human oocytes. Very recently a GWAS approach on a US DS [36] sample has identified some of the genomic loci that exhibited distinct associations either with MI or with MII NDJ. All these candidate genes can also be tested following our present work for their association with meiotic exchange events in interaction with maternal age at conception. The effect of the maternal MCM9 risk variants on the recombination profile of normally segregating Ch21 among the euploid siblings of individuals with DS is of further scientific interest. To gain more insight into the role of MCM9 and other associated factors in the etiology of Ch21 missegregation, replication of the work in other populations is warranted.

## Supporting information

S1 Table Summary of the outcome of *in silico* analysis of the twenty five risk variants using bioinformatics tools PolyPhen 2, *Mutationt@ster*, PROVEAN and SIFT shows respective scores. (XLSX)

S2 Table Summary of the outcome of *in silico* analysis of all twenty five MI risk variants on splicing of MCM9 transcript using Human Splice Finder (HSF) program with estimated score for each polymorphic site. (XLSX)

S3 Table List of primers used for analyses of exonic sequence in MCM9 gene. (XLSX)
